# An update to experimental and clinical aspects of tumor-associated macrophages in cancer development: hopes and pitfalls

**DOI:** 10.1007/s10238-024-01417-w

**Published:** 2024-07-13

**Authors:** Arash Salmaninejad, Sepideh Mehrpour Layeghi, Zeinab Falakian, Shahin Golestani, Sepehr Kobravi, Samaneh Talebi, Meysam Yousefi

**Affiliations:** 1 Department of Medical Genetics, School of Medicine, Mashhad University of Medical Sciences, Mashhad, Iran; 2 Department of Medical Genetics, School of Medicine, Tehran University of Medical Sciences, Tehran, Iran; 3 Pediatric Diseases Research Center, Guilan University of Medical Sciences, Rasht, Iran; 4 Department of Laboratory Science, Lahijan Branch, Islamic Azad University, Lahijan, Iran; 5 Department of Ophthalmology, School of Medicine, Shahid Beheshti University of Medical Sciences, Tehran, Iran; 6 Department of Oral and Maxillofacial Surgery, Tehran Azad University, Tehran, Iran; 7 Department of Medical Genetics, Faculty of Medicine, Ahvaz Jundishapur University of Medical Sciences, Ahvaz, Iran

**Keywords:** Tumor-associated macrophages, Cancer development, Tumor microenvironment, Metastasis

## Abstract

Tumor-associated macrophages (TAMs) represent one of the most abundant tumor-infiltrating stromal cells, and their normal function in tumor microenvironment (TME) is to suppress tumor cells by producing cytokines which trigger both direct cell cytotoxicity and antibody-mediated immune response. However, upon prolonged exposure to TME, the classical function of these so-called M1-type TAMs can be converted to another type, “M2-type,” which are recruited by tumor cells so that they promote tumor growth and metastasis. This is the reason why the accumulation of TAMs in TME is correlated with poor prognosis in cancer patients. Both M1- and M2-types have high degree of plasticity, and M2-type cells can be reprogrammed to M1-type for therapeutic purposes. This characteristic introduces TAMs as promising target for developing novel cancer treatments. In addition, inhibition of M2-type cells and blocking their recruitment in TME, as well as their depletion by inducing apoptosis, are other approaches for effective immunotherapy of cancer. In this review, we summarize the potential of TAMs to be targeted for cancer immunotherapy and provide an up-to-date about novel strategies for targeting TAMs.

## Introduction

The tumor microenvironment (TME) consists of a diverse combination of chemicals and cells that surround tumor cells as a multifaceted organ composed of non-cancerous stromal cells, blood vessels, lymphatic vessels, extracellular matrix (ECM) and secretory proteins [1, 2]. A major part of stromal cells in TME includes immune cells and substances such as dendritic cells, natural killer (NK) cells, regulatory T cells and macrophages scattered in the cancerous mass. The functions and roles of these compounds are different based on the degree and category of tumor progression [3].

Macrophages are a type of versatile immune cells responsible for regulating tissue homeostasis, fighting infections, and contributing to wound healing and other functions. Although it is now widely accepted that most macrophages originate from monocytes, the exact origin of macrophages is unclear because tissue-resident macrophages such as alveolar macrophages in the lungs, brain microglia, and Kupffer cells in the liver are not derived from blood monocytes and substitute for the origin mechanisms self-renewal and proliferation of these cells have not been identified [4].

Macrophages are the most abundant immune cells in the TME and after infiltrating into tumor mass, they are referred to as tumor-associated macrophages (TAMs) [5]. The normal function of TAMs is to suppress tumor formation. This function is mediated through different mechanisms, including direct phagocytosis of tumor cells, the induction of T cell-mediated cell cytotoxicity and the stimulation of antibody-mediated immune response. This type of TAMs is usually referred to as M1-type. However, tumor cells can evade the antineoplastic properties of TAMs through different mechanisms. For example, the cluster of differentiation 47 (CD47), known as the “do-not-eat-me” signal is widely expressed on the membrane of tumor cells. The ligand of CD47, signal regulatory protein alpha (SIRPα), is mainly expressed on macrophages and when the interaction with CD47 occurs, the downstream signaling within tumor cells results in the dephosphorylation of multiple substrates, keeping tumor cells from phagocytosis by macrophages [6]. After evading phagocytosis, tumor cells can further recruit TAMs to help them better proliferate and disseminate throughout the body. This second type of TAMs with compromised characteristic is called M2-type [7].

**Fig. 1:**
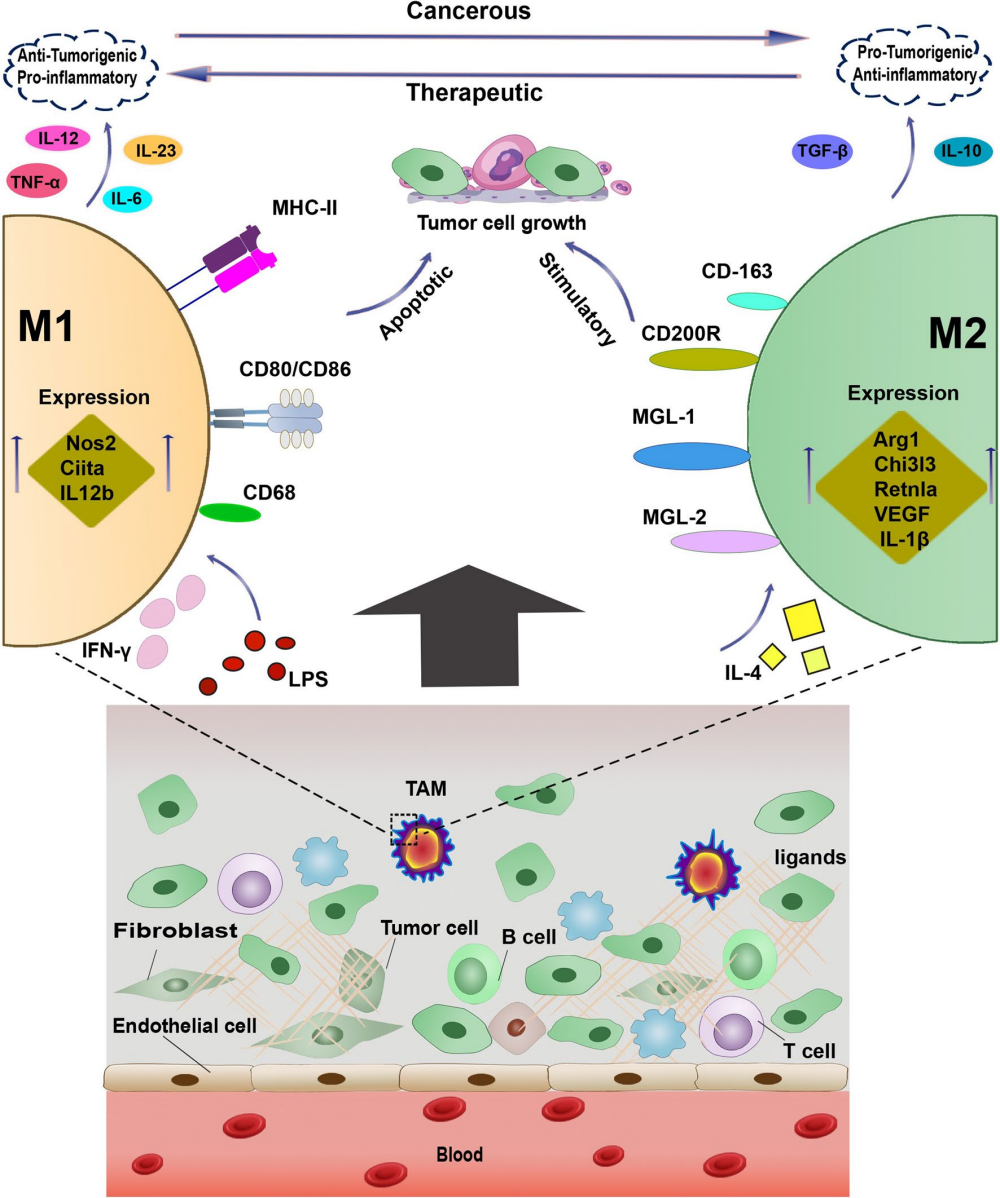
The transition between M1 and M2 TAMs. The normal function of TAMs is to suppress tumor formation, which is mediated through different mechanisms, including direct phagocytosis of tumor cells, the induction of T cell-mediated cell cytotoxicity and the stimulation of antibody-mediated immune response. This type of TAMs is usually referred to as M1-type and respond to harmful signals sent by IFN-γ and exhibit heightened expression of Ciita, iNOS, ROS and IL-12, which primarily possess antitumor effects. Some tumor cells can evade from M1-type mediated phagocytosis and further recruit TAMs to help them better proliferate and disseminate throughout the body. This second type of TAMs with compromised characteristic is called M2-type. M2 macrophages express high levels of VEGF, IL-10, IL-1β, Chi3l3, Arg1, and Retnla, which contribute to tumor cell growth, angiogenesis and metastasis

M1 cells typically respond to harmful signals sent by interferon γ (IFN-γ) and exhibit heightened expression of inducible nitric oxide synthase (iNOS), reactive oxygen species (ROS), and interleukin 12 (IL-12), which primarily possess antitumor effects and aid in distinguishing tumor cells from normal cells [8, 9]. On the other hand, M2 macrophages express high levels of VEGF, IL-10, IL-1β, and matrix metalloproteinases (MMPs), which contribute to chemoresistance, tumor angiogenesis and metastasis [10, 11] (Fig. 1). The accumulation of M2-type TAMs is often linked to a poor prognosis in human cancers including lung, breast, and gastric cancers [12, 13].

In recent years, the traditional M1/M2 polarization model and other macrophage nomenclature systems have been challenged as they are not satisfactory enough to address the heterogeneity of macrophages. In this regard, a group of macrophage specialists, congregated at the International Congress of Immunology in Milan, recommended some modifications and suggested a spectrum concept for the activation of macrophages according to the stimulation conditions [14]. Subsequent studies revealed that following stimulation with various activation signals macrophages can be categorized in several groups, based on their transcriptome and proteome profiles. For instance, Xue et al. analyzed a dataset of 299 human macrophage transcriptomes and their network modeling suggested that at least nine distinct groups are distinguishable in a spectrum in which each particular phenotype was associated with distinct transcription factors [15]. The novel spectrum concept will bring new strategies for targeting TAM.

Single-cell RNA sequencing (scRNA-seq) is a powerful technique that scrutinizes individual cell transcriptomes, unveiling the varied gene expression across individual TAMs to identify their heterogeneity. This technology is influential in exploring the mechanisms supporting tumor progression and therapeutic strategies [16]. Current scRNA-seq investigations predominantly focus on some solid tumors like glioma, breast cancer, and lung cancer [17–19]. In various solid tumor tissues, functional enrichment analysis and gene expression profiling in scRNA-seq data delineate distinct roles for different tumor-associated macrophages (TAMs): FCN1 + TAMs show a proinflammatory influence, SPP1 + TAMs are implicated in metastasis, angiogenesis, and cancer stem cell activation, C1Q + TAMs play a role in immune regulation and suppression, and CCL18 + cells represent terminal immunosuppressive macrophages with potent immunosuppressive abilities and the capacity to promote tumor metastasis[20]. By studying TAM heterogeneity, scRNA-seq explains the correlation between tissue-specific macrophage subtypes and tumor progression within the tumor microenvironment, conveying valuable insights for patient prognosis. It also identifies novel cellular and molecular markers that hold promise as targets for cancer therapy [21].

Regarding the dual potential of TAMs as antineoplastic and cancer-stimulating cells, and the high degree of plasticity these immune cells show, TAMs are currently regarded as intriguing targets for cancer immunotherapy. For instance, negative regulation of CD47/SIRPα signaling can confer antitumor properties to TAMs and reprogram them into M1-type with high phagocytic capacity. In addition, given the correlation between the density of TAMs and cancer prognosis, blocking the recruitment of macrophages in TME, for instance through ablation of CCL2/CCR2 axis, has been shown to be an effective therapeutic strategy to reduce the density of protumor TAMs and improve prognosis [22, 23]. Furthermore, depletion of TAMs by inducing apoptosis, which can be obtained through targeting CSF-1/CSF-1R signaling, can regulate the proliferation of TAMs within TME and impede their transition from M1-type to M2-type [24]. In this article, we aim to review the potential of TAMs to be targeted for cancer immunotherapy and explain the molecular mechanisms underlying this approach.

## Inhibition of protumor action of TAMs

**Fig. 2:**
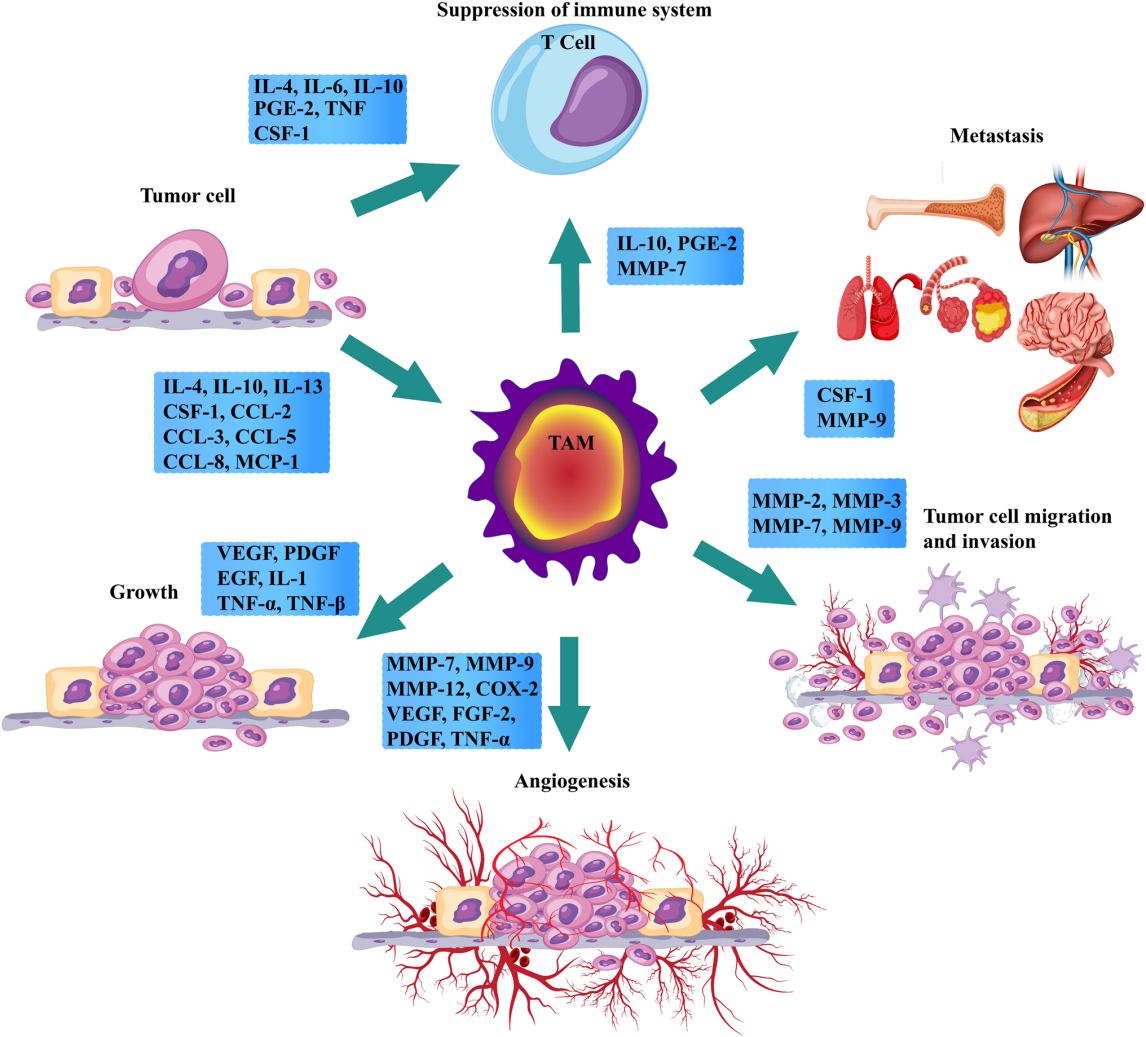
Various effects of TAMs on tumor cell invasion and metastasis. TAMs can stimulate tumor cell growth, tumor cell migration, angiogenesis and metastasis to distant organs. They can also suppress immune system through decreasing CD8 + T cells, resulting in markedly depletion of macrophages and diminishing tumor cell phagocytosis

Numerous cytokine and chemokine stimuli contribute to monocyte/macrophage recruitment to the TME, among which are CCL2 and C-X-C motif chemokine ligand 12 (CXCL12). CCL2 together with its receptor, CC-motif chemokine receptor 2 (CCR2) are crucial for mobilization and recruitment of bone marrow-derived monocytes into solid tumors [25]. Blocking the recruitment of macrophages in TME has been shown to be an effective therapeutic strategy to reduce the density of protumor TAMs and improve prognosis. Ablation of CCL2/CCR2 axis via neutralizing monoclonal antibodies (mAbs), antisense RNA or genetic manipulation has been shown to inhibit the development of tumor progression and metastasis in bone marrow, breast, liver and lung cancers [22, 23, 26, 27]. Moreover, studies on animal models demonstrated that CCL2/CCR2 blockade in combination with traditional chemotherapy or immunotherapy can synergize the antitumor effects [23]. However, there are still concerns regarding the possibility of metastasis exacerbation following therapy cessation which need to be further investigated [23]. In a study on a mouse model of breast cancer, for instance, the cessation of therapy which was based on blockade of CCL2/CCR2 axis, resulted in exacerbation of lung metastasis. This was linked to elevated monocyte release and promoting angiogenesis in cancer cells [28]. Carlumab (CNTO888), an antibody which targets CCL2, and PF-04136309, a molecular agent with inhibitory effects on CCR2, are under clinical trials showing preliminary promising results in solid tumors [29, 30]. CXCL12 and its receptor; C-X-C motif chemokine receptor 4 (CXCR4) are involved in recruitment of tumor-promoting macrophages. This is because CXCL12 is able to activate monocytes-derived immunosuppressive macrophages with lower capability of T lymphocyte stimulation which in turn results in enhanced migration and accumulation of macrophages in cancer cells. Therefore, disrupting CXCL12/CXCR4 axis can be a potential therapeutic modality in preventing the recruitment of TAMs into tumors. Using this therapeutic approach led to the prolonged survival and reduced tumor burden in murine ovarian and prostate models [31]. Moreover, targeting C-X3-C motif chemokine ligand 1 (CX3CL1) and C-X3-C motif chemokine receptor 1 (CX3CR1), which are involved in mobilization of tumor-promoting macrophages, can also be an effective therapeutic intervention with possible effects in disrupting the recruitment of immune cells in preclinical models [32]. Taken together, disrupting TAM prorecruitment signaling pathways can be effectively used to hinder tumor growth and progression in solid tumors. TAMs may display both tumor growth endorsing and hindering activities. They may help tumor growth not only through stimulating angiogenesis, but also through suppressing acquired immune responses (Fig. 2).

## Depletion of TAMs

Depletion of TAMs by inducing apoptosis is a promising approach for effective immunotherapy of cancer [23]. A prominent example is targeting of CSF-1 and its receptor (CSF-1R) which contribute to regulation of macrophage proliferation and survival as well as transition of TAM M1 into TAM M2 type [24]. Upregulation of CSF-1 and CSF-1R is linked to poor prognosis in several types of tumors [33]. Blocking this signaling axis can lead to apoptosis in a significant portion of tumor-stimulating TAMs. The antitumor effect of CSF-1R inhibitor pexidartinib (PLX3397) is believed to be associated with reduced CD4^+^ T cells and elevated CD8^+^ T cells, resulting in markedly depletion of macrophages and inhibition of tumor growth [23]. This drug is the first and most effective immunotherapeutic agent to show a promising response in patients with tenosynovial giant cell tumor (TGCT) [34]. Emactuzumab, a mAb for CSF-1R, also showed promising results with no significant toxicity when used at the optimal immunomodulatory doses [35]. Cabiralizumab (FPA008) is another drug which inhibits CSF-1 and IL-34 from binding to CSF-1R, thereby interrupting the pathway. Cabiralizumab has been used in phase I trials of advanced solid tumors [36, 37]. Several other mAbs, tyrosine kinase inhibitors, small molecules and compounds targeting CSF-1/CSF-1R pathway are under development as single agents or in combination in order drugs to deplete TAMs by inducing their apoptosis. Trabectedin, for instance, has been shown to kill tumor-stimulating macrophages through induction of DNA damage and cell cycle arrest [33, 38]. Bisphosphonate compounds, including zoledronic and clodronate, are also proapoptotic and absorbed by highly phagocytic macrophages which in turn inhibit their proliferation [39]. Bisphosphonate treatments have been shown to be effective in breast cancer resulting in reduced neovascularization, macrophage depletion and improvement of survival [22]. In terms of unwanted adverse effects, both mAbs and tyrosine kinase inhibitors are considered to be well tolerated with no significant clinical consequences. However, the most common adverse events include elevated liver enzymes, pruritus, edema, asthenia and fatigue [40]. Similar to recruitment-inhibitory agents, depletion-causing agents are also thought to be more effective and better tolerated if they are prescribed in combination with conventional therapies.

## Reprogramming TAMs to induce antitumor activity

In early neoplastic tissues, macrophages exert their positive antitumor roles and impede tumor growth. However, prolonged exposure of macrophages to TME during progression of malignancies can trigger their tumorigenic properties. This indicates that antineoplastic effects of macrophages can be restored due to their plasticity [22]. Besides, extreme therapeutic depletion of TAMs may bring side effects such as chronic inflammation. Therefore, reprogramming tumorigenic TAMs to convert them into cells with tumoricidal characteristics has drawn extensive attention [30, 41]. CD47, known as the “do-not-eat-me” signal is widely expressed on membrane of various cells including tumor cells. The ligand of CD47, SIRPα, is mainly expressed on macrophages and when their interaction occurs, its downstream signaling within tumor cells results in the dephosphorylation of multiple substrates, keeping tumor cells from phagocytosis by macrophages [6]. Negative regulation of CD47/SIRPα signaling can bestow antitumor properties to TAMs and restore their phagocytic capacity. CD47/SIRPα disruption can be achieved by anti-CD47 and/or anti-SIRPα antibodies or even engineered receptors mimicking the interaction with no triggering of downstream signaling [6]. SIRPα protein ALX148 and SIRPα-Fc fusion protein (TTI-621) are examples of engineered molecules with therapeutic goals [42]. This therapeutic strategy has been proven effective in several human cancers [43, 44]. TTI-621 has been shown to be effective in enhancing phagocytosis of hematologic as well as solid tumors because of blocking CD47/SIRPα signaling [43, 45]. CD47 antibody Hu5F9-G4 showed enhancement of phagocytic potential of macrophages and suppression of tumor cells in small cell lung carcinoma (SCLC) [46].

Programmed cell death protein (PD-1) and its ligand, programmed cell death-ligand 1 (PD-L1) also play a role in keeping tumor cells from phagocytosis. PD-1/PD-L1 signaling pathway can help tumor cells escape the immune response due to diminished functions of TAMs and other immune cells. Thus, blocking this signaling axis has the potential to improve phagocytic activity of TAMs and hinder tumor progression [47]. MHC class I component β2-microglobulin which is expressed on the surface of tumor cells, and its receptor, leukocyte immunoglobulin like receptor subfamily B member 1 (LILRB1), expressed on membrane of macrophages provides another therapeutic target. Blocking of β2-microglobulin can induce the phagocytic capability of recognizing macrophages. In addition, downregulation or knockout of LILRB1 can convert macrophages in order to limit tumor growth [48]. CD40, which is expressed by both tumor cells and macrophages can cause the release of proinflammatory cytokines as well as CD80 and CD86 thereby resulting in augmentation of ant-tumor functions [49]. The activation of CD40 or using its agonists can therefore restore tumoricidal properties of macrophages [50]. Other therapeutic strategies to reprogram TAMs include but not limited to inhibitors of phosphatidylinositol 3-kinase (PI3K), class II histone deacetylases (HDACs) and CD24/siglec-10 [51, 52]. Depending on the microenvironment, macrophages show very diverse phenotypes. M1 macrophages are activated with lipopolysaccharide (LPS) and IFN-γ and identified by the expression of Ciita, Nos2, and IL12b, whereas M2 macrophages are activated with IL-4 and characterized by the expression of Chi3l3, Arg1, and Retnla [53]. M1 macrophages promote inflammatory responses against pathogens and tumor cells, whereas anti-inflammatory characteristics of M2 macrophages are involved in wound healing and tumor progression (Fig. 1).

Induction of tumoricidal effects of TAMs through their reprogramming has drawn extensive attention and can provide novel anticancer therapies. Pattern recognition scavenger receptor MARCO defines a subtype of suppressive TAMs is linked to clinical outcome. An anti-MARCO monoclonal antibody was developed, which makes antitumor activity in colon and breast carcinoma, along with in melanoma models over reprogramming TAM populations to a proinflammatory phenotype and enhancing tumor immunogenicity. This antitumor activity is reliant on the inhibitory Fc-receptor, FcγRIIB, as well increase the efficacy of checkpoint therapy [54].

## TAMs as a therapeutic target in angiogenesis

A wide variety of angiogenic stimulators and inhibitors regulate abnormal blood vessel formation in tumors by affecting endothelial cell (EC) migration, proliferation, and differentiation [55]. Since tumorigenesis is mainly dependent on neo-angiogenesis, inhibition of angiogenesis is considered as one of the most effective treatment strategies in various solid tumors, including breast cancer, lung cancer, colorectal cancer (CRC), etc. [56–58]. TAMs, are a major type of inflammatory cells comprising part of stromal cells in TME which control the angiogenesis processes [56]. Besides, a significant correlation has been identified between the number of TAMs and the density of blood vessels in human tumor specimens, including glioma and breast cancer [59]. TAMs have been found to secrete a variety of proangiogenic factors, including tumor-necrosis-factor α (TNFα), MMPs, e.g., MMP2, MMP9, and MMP12, serine or cysteine proteinases, such as cathepsins, plasminogen activator (PA), thymidine phosphorylase, as well as growth factors such as angiopoietin 1 and 2 (Ang-1 and Ang-2), VEGF, epidermal growth factor (EGF), transforming growth factor-α and -β (TGF-α and TGF-β), and platelet-derived growth factor (PDGF) which can promote angiogenesis in various cancers [56, 59–61]. TAMs have also been shown to release extracellular matrix (ECM) remodeling mediators that degrade ECM to promote sustained activation and proliferation of ECs [60]. The proangiogenic effects of TAMs involve the coordinated regulation of a wide variety of inflammatory cytokines, including IL-1, IL-6, IL-8, CXC-chemokine ligand 8 (CXCL8), cyclooxygenase 2 (COX2), and nitric oxide (NO) [60, 62]. These are among the main pathways and factors to be targeted by antiangiogenetic molecules.

Several strategies can be used to inhibit angiogenesis. These strategies include the application of inhibitors of metalloproteinases, mAbs against growth factors or their receptors, and signal transduction inhibitors [63]. TAM inhibitors mainly consist of inhibitors of VEGF/angiopoietin, PI3Kγ, CSF-1R, and IPI-549 [55, 60, 64]. TAM-derived VEGF and its receptors have been found to play major roles in regulating and promoting tumor angiogenesis [55, 60, 64]. VEGF can act as a negative regulator of the TME and stimulator of TAM accumulation [60]. The humanized mAb bevacizumab (Avastin), specific for VEGF, was the first FDA-approved antiangiogenic agent for first-line treatment of metastatic CRC in combination with chemotherapy [60, 64]. As of February 2021, bevacizumab has also been approved in Japan for six types of malignancies including advanced or recurrent non-small cell lung cancer (NSCLC excluding squamous cell carcinoma), advanced or recurrent cervical cancer, advanced or recurrent unresectable CRC, high-grade gliomas, unresectable or recurrent breast cancer, and ovarian cancer [52]. Bevacizumab and aflibercept can cause a decrease in VEGFA effects and an increase in CD206 + and Tie-2 + macrophages in murine and human gliomas through increasing Ang2 levels [57]. In addition to bevacizumab, numerous VEGF and VEGFR inhibitors, including multi-kinase inhibitors, such as sunitinib and sorafenib, have been found to be an effective strategy to attenuate angiogenesis by inducing apoptosis of ECs [55]. Moreover, axitinib, pazopanib, vatalanib, the VEGF-targeted fusion protein Afibercept, and the anti-VEGFR2 antibody ramucirumab are also under clinical investigation for treatment of high-grade gliomas [60]. Besides, VEGFA secretion by TAMs stimulates neovascularization and relapse, which could be inhibited by the administration of CXCR4 inhibitor AMD3100 [65]. The pan-VEGFR inhibitor, Cediranib, transiently reduces the intratumoral infiltration of macrophages. However, it increases the serum level of VEGFA and the number of CXCR4- and CD45-positive circulating cells in the peripheral blood, thereby increasing angiogenesis in mouse models of pancreatic neuroendocrine and breast tumors. Cediranib stimulates STAT3 signaling in macrophages, which triggers angiogenesis through VEGFA production [57]. A specific JAK-STAT inhibitor, AZD1480, counteracted adverse effects of STAT3 activation in TAMs in a murine xenograft glioma model [66]. Likewise, the sunitinib-induced antiangiogenic effects were increased by simultaneous treatment with STAT3 inhibitors in a metastatic renal cell carcinoma model. The intravitreal drug ranibizumab blocks VEGFR1 phosphorylation and reduces the migratory capacity of TAMs in experimental colon cancer, leading to a reduction in the number of TAMs and tumor angiogenesis [57].

Since anti-VEGF/VEGFR monotherapy is not able to completely suppress tumor angiogenesis, treatment strategies considering multiple perspectives and approaches other than anti-VEGF/VEGFR monotherapy are essential to effectively overcome angiogenesis-induced tumor progression [60]. In angiogenesis-based treatments, resistance to treatment are frequently seen, which can be managed by administrating angiogenesis inhibitors in combination with chemotherapy, novel target drugs, or immune checkpoint blockades [64]. Metastatic melanoma has recently treated with bevacizumab combined with the anti-CTLA4 ipilimumab, an immune checkpoint inhibitor showed an effective therapy response [64]. Moreover, olaparib plus the TKI cediranib significantly improved progression-free survival (PFS) in patients with endometrial ovarian cancer or platinum-sensitive high-grade serous compared with patients treated with olaparib alone. Ovarian and cervical cancers also show sensitivity to VEGF inhibitors, but mainly with co-administered chemotherapy, novel classes of target drugs, or drugs with immunomodulatory properties [60, 67]. The combination of bevacizumab and lomustine in patients with recurrent glioblastoma improved PFS compared to monotherapy, suggesting an additive/synergistic effect of bevacizumab on immunotherapy [55]. Therefore, it is suggested that a combination of antiangiogenic therapies targeting VEGF, including bevacizumab and immunotherapy, could be a potentially effective therapeutic strategy. Various mAbs for targeting TAMs or its factors are listed in Table 1.

**Table 1:** Various therapeutics designed for targeting tumor-associated macrophages in human cancers

Class of drug or category	Drug or substance	Target	Compound characteristics	Study/clinical trials or Ref	Tumor type	Trial design	Treatment
Mono clonal AB or blocker	Emactuzumab (RG715)	CSF1R	Blockade of colony-stimulating factor-1 receptor (CSF-1R) enables the therapeutic targeting of tumor-associated macrophages (TAM)	NCT02760797	Neoplasms of solid tumors	Phase I	Agonistic anti-CD40 mAb selicrelumab
				NCT02923739	Primary peritoneal cancer	Phase II	Paclitaxel + anti-VEGFA mAb bevacizumab
				NCT01494688	Solid tumors	Phase I	Monotherapy; paclitaxel
	Cabiralizumab (FPA008)	CSF1R	Binds to CSF1R expressed on monocytes, macrophages, and Oste oclasts and inhibits CSF1R ligands colony-stimulating factor-1 (CSF-1) and interleukin-34 (IL-34), from binding to CSF1R	NCT01346358	Neoplasms of solid tumors	Phase I	IMC-CS4
				NCT03101254	Melanoma	Phase I/II	Vemurafenib, Cobimetinib
	IMC-CS4	CSF1R	A monoclonal Antibody Targeted to the CSF-1 Receptor (CSF-1R)	NCT04331067	Breast cancer	Phase I/II	Paclitaxel Carboplatin Nivolumab Cabiralizumab
				NCT00992186	Prostate cancer	Phase II	Carlumab
	AMG820	CSF1R	Inhibits binding of CSF1 and interleukin (IL)-34	NCT02225002	Pancreatic cancer, CRC, NSCLC	Phase I/II	Pembrolizumab
	LY3022855	CSF1R	Leads to TAM apoptosis by inhibiting CSF-1R signaling,	NCT02953782	Melanoma	Phase I/II	MEK/BRAF inhibitors
	Cabiralizumab	CSF1R	A humanized IgG4 monoclonal antibody, binds to CSF-1R and blocks its signaling, a key determinant of TAM activation and survival	NCT03990233	TNBC	Phase I/II	Paclitaxel, carboplatin, nivolumab
	Imalumab	MIF	A recombinant, human IgG1 monoclonal antibody that targets macrophage inhibitory factor (MIF)	NCT01765790	Solid tumors	Phase I	Monotherapy
	ANTI CD5L	CD5L	Monoclonal antibodies (mAbs) against recombinant CD5L	[68]	Papillary lung adenocarcinoma	–	–
	*Carlumab*	CCL2	A human immunoglobulin G1κ monoclonal antibody that specifically binds and neutralizes profibrotic activities of human CCL2	NCT00992186	Prostate cancer	Phase II	Monotherapy
	*SEA-CD40*	CD40	A nonfucosylated, receptor-agnostic, humanized IgG1 CD40-directed monoclonal antibody that binds with increased affinity to FcγRIIIa, resulting in enhanced effector function	NCTR20200938	Solid and haematological tumors	Phase I	Pembrolizumab, gemcitabine, Nab-paclitaxel
	CP-870,893	CD40	A fully human, IgG2 antibody that selectively interacts with CD40 at a site distinct from its ligand-binding region	NCT04495257	Advanced melanoma or renal cell carcinoma (RCC)	Phase I	Nivolumab and Ipilimumab
	APX005M	CD40	*A* humanized IgG1 monoclonal antibody that binds CD40 with high affinity and blocks the binding of CD40 to CD40L	NCT03733990	Melanoma, renal carcinoma	Phase I	Nivolumab, ipilimumab
	RO7009789	CD40	A recombinant fully human monoclonal antibody of the IgG2 subclass with agonist activity on the CD40 receptor	NCT02304393	Solid tumors	Phase I	Atezolizumab
	CDX-1140	CD40	A human IgG2 antibody that activates DCs and B cells and drives NFkB stimulation in a CD40-expressing reporter cell line	NCT04675294	Breast cancer	Phase I	Radiation, biological therapy, poly-ICLC
	NG-350A adenoviral vector	CD40	An oncolytic *adenovirus vector* expressing a CD40 monoclonal antibody that activates the immune system for an antitumor response	NCT02663518	Solid tumors	Phase I	Immune-checkpoint blockade immunotherapy
	Hu5F9-G4	CD47- SIRP-a	An antibody which is an immune checkpoint inhibitor blocking CD47 that induces tumor cell phagocytosis	NCT02665416	Solid tumors	Phase I/II	Cetuximab
	BI 754091	CD47	A monoclonal IgG4Pro antibody inhibitor of PD-1 with proven in vitro and in vivo antitumor activity	NCT04616248	Solid tumors	Phase I	BI 754091 (anti-PD1)
	IBI188	CD47	A humanized IgG4 monoclonal antibody targeting CD47	NCT05165433	advanced malignant tumors	Phase I	chemotherapy
	IBI188	CD47	A humanized IgG4 monoclonal antibody targeting CD47	NCT04691375	acute myeloid leukemia	Phase I	PD-1 inhibitor chemotherapy

	*ALX148*	CD47	ALX148 works by blocking a protein called CD47	NCT04675294	Head and neck squamous cell carcinoma (HNSCC)	Phase II	Pembrolizumab,
	TTI-621 (Trillium)	CD47	Triggers phagocytosis of lymphoma cells by M1-like and M2-like tumor-associated macrophages	NCT02663518	Hematological malignancies	Phase I	Nivolumab, rituximab
	*AK117*	CD47- SIRP-a	*AK117* is a novel humanized IgG4 monoclonal antibody (mAb) targeting CD47	NCT01525602	advanced solid tumors/lymphomas	Phase I	Monotherapy
	FP-1305	Clever 1	*FP*-*1305* is a novel IgG4-antibody targeting CLEVER-1 and induces a phenotypic M2 to M1 immune switch of TAMs.	NCT03733990	Solid tumors	Phase I	monotherapy
	IPH5401	C5aR	*IPH5401*, a fully human anti-C5aR1 antibody, inhibits the C5a mediated effects on MDSC and neutrophils	NCT02071940	Solid tumors	Phase I	Durvalumab
	BDC-1001	TLR7/8	An immune stimulating antibody conjugate (ISAC), which conjugates Toll-like receptors (TLRs) with tumor-targeting antibodies for localized immune-stimulation at the tumor site	NCT04278144	HER2^+^ solid tumors	Phase I/II	Pembrolizumab
	Carlumab	CCl2	A recombinant human IgG1 monoclonal antibody targeting CC chemokine ligand 2 (CCL2)	NCT00992186	Prostate cancer	Phase II	Monotherapy
	MEDI3617	Ang2	Anti-Ang2 mAb inhibiting Ang2 binding to TIE2	NCT01248949	Advanced solid tumors*.*	Phase I	Monotherapy; paclitaxel; paclitaxel + carboplatin; bevacizumab
	Vanucizumab (RG7221)	VEGF/ Ang2	Anti-VEGF/Ang2 bispecific mAb inhibiting neoangiogenesi	NCT01688206	Advanced ovarian cancer	Phase I	Monotherapy; anti-PD-L1 mAb atezolizumab
	PY314	TREM2	Anti-TREM2 mAb depleting TREM2^+^ TAMs through ADCC and/or ADCP	NCT04691375	Various cancers	Phase I	Pembrolizumab
	BI-765063	SIRPα	Antagonistic anti-SIRPα mAb	NCT03990233	Solid tumor,	Phase I	Monotherapy
	JTX8064	LILRB2	JTX-8064 is a humanized IgG4 monoclonal antibody that specifically binds to macrophage receptor LILRB2/ILT4	NCT04669899	Advanced refractory solid tumors	Phase I	Pimivalimab
	Anti-MARCO	MARCO	Suppressing the immunosup- pressive TME	[54]	Breast and colon carcinoma	–	–
	Epacadostat	IDO	antagonistic anti-SIRPα mAb	NCT01685255	Ovarian cancer genitourinary (GU) tumors	Phase II	Monotherapy

Tyrosine kinase inhibitors	Pexidartinib (PLX3397	CSF1R CKIT, FLT3, PDGFR	Pexidartinib binds to and inhibits phosphorylation of stem cell factor receptor (KIT), colony-stimulating factor-1 receptor (CSF1R) and FMS-like tyrosine kinase 3 (FLT3	NCT02880371	Advanced solid tumors	Phase I/II	Pembrolizumab, ARRY-382
				NCT01804530	Tumors of any histology with activating Trk (NTRK) point or NTRK fusion mutations tenosynovial giant cell tumor	Phase 1	Monotheapy
				NCT02829723	Advanced solid tumors	Phase I/II	Monothrapy
	ARRY-382	CSF1R	A small molecule and orally available inhibitor of CSF-1	NCTR20160572	Advanced solid tumors refractory to standard therapy	Phase I	Monothrappy
	PLX7486	CSF1R TRKA-B-C	A selective inhibitor of the receptor tyrosine kinases colony-stimulating factor-1 receptor (CSF1R; fms) and neurotrophic tyrosine kinase receptor types 1, 2 and 3 (TrkA, TrkB, and TrkC	NCT03177460	Advanced solid tumors refractory to standard therapy	Phase I	Monothrapy
	BLZ945	CSF1R	BLZ945 selectively binds to CSF1R expressed on TAMs, blocks CSF1R activity, and inhibits CSF1R-mediated signal transduction pathways	NCT01054014	Advanced solid tumors refractory to standard therapy	Phase I/II	combined with PDR001
	Sulfatinib	CSF1R	*Sulfatinib* is a multi-targeted TKI that targets FGFR1, SF1R, and vascular endothelial growth factor receptors (VEGFR1–3)	NCT03866109	Thyroid carcinoma	Phase II	Monoterapy
				NCT04660929	Advanced cancers	Phase I	CT-0508 Pembrolizumab
	JNJ- 0,346,527	CSF1R	A small molecule, orally available inhibitor of colony-stimulating factor-1 receptor (CSF1R; FMS) with potential antineoplastic activity. FMS tyrosine kinase inhibitor JNJ-40346527 blocks the receptor-ligand interaction between FMS and its ligand CSF 1	NCT01413022	Pancreatic cancer	Phase I	Oxaliplatin Irinotecan: Leucovorin Fluorouracil
	Eganelisib (IPI-549)	PI3Kγ	Selective PI3Kγ inhibitor reprogramming key immune suppressive cells	NCT02637531	Advanced solid tumors	Phase I	Monotherapy; nivolumab

Biologicall response modifier	TEMFERON	Macrophage	Temferon release the known antitumor agent IFN-α by altering Tie-2-expressing monocytes, which restores the immune system and offsets tumor growth without the high amount of toxicity that IFN-α is associated with as a single-agent	NCT04660929	Glioblastoma	Phase I/II	Monotherapy
	CT-0508	Macrophage	A cell product comprised of autologous monocyte-derived proinflammatory macrophages expressing an anti-HER2 CAR	NCT04660929	HER2^+^ solid tumors	Phase I	Pembrolizumab
	BMS-813160	CCR2/CCR5	*A* potent and selective CCR2/5 dual antagonist; binds to CCR2 and CCR5	NCT04588324	Renal carcinoma	Phase II	Nivolumab plus ipilimumab
	PF-4136309	CCR2	An orally available human chemokine receptor 2 (CCR2) antagonist with potential immunomodulating and antineoplastic activities	NCT04278144	Solid tumors	Phase II	Nab-paclitaxel, gemcitabine
	CCR5 antagonist	CCR5	a class of small molecules that antagonize the CCR5 receptor	NCT03274804	CRC	Phase I	Pembrolizumab
	BO-112	TLR3	Acts by activating TLR3, RIG-1 and MDA-5, leading to an immunogenic cell death and increasing immune checkpoint inhibition effects	NCT04508140	CRC, gastric cancer	Phase II	Pembrolizumab
	SHR2150	TLR7	A TLR7 selective agonist	NCT04588324	Solid tumors	Phase I/II	Anti-PD1 and/or anti-CD47
	TransCon	TLR7/8	*TransCon TLR7/8 Agonist* is a long-acting prodrug of resiquimod, a small molecule agonist of TLR7 and TLR8	NCT04799054	Solid tumors	Phase I/II	Pembrolizumab
	CMP-001 (	TLR9	A first-in-class CpG-A oligonucleotide that activates the innate immune system via TLR9	NCT03084640	Melanoma	Phase I	Pembrolizumab
	Dual-inhibitor-loaded nanoparticles (DNTs)	M2 macrophages	*Make M2 macrophages repolarize to active M1* *macrophages and inhibit CSF1R and SHP-2*	[69]	Breast cancer and melanoma mouse models	–	–
	α-Difluoromethylornithine (DFMO)	Macrophages	*Regulate of macrophages polarization*	[70]	Murine ovarian cancer	–	–
	DNMTi 5-Azacytidine (AZA)	Macrophages	*Regulate of macrophages polarization*	[71]	Murine ovarian cancer	–	–
	GEN-1 (IP IMNN-001)	IL-12	L-12 plasmid/lipopolymer complex stimulating the antitumor immune responses	NCT02480374	Epithelial Ovarian Cancer (EOC) Fallopian Tube Cancer Primary Peritoneal Cancer	Phase I	Neoadjuvant paclitaxel + carboplatin
	CT-0508	HER2	CAR macrophages targeting HER2 + tumor cells	NCT04660929	Neoplasms	Phase I	Monotherapy
	NI-1801	CD47/mesothelin	NI-1801 is a human IgG1 bispecific antibody targeting MSLN and CD47	NCT05403554	Epithelial ovarian cancer, TNBC, NSCLC	Phase I	Monotherapy
Targeting TAMs metabolism	2-DG (Glucose metabolism)	Hexokinase 2	Targets glucose metabolism to deplete cancer cells of energy	[72]	Human pancreatic ductal adenocarcinoma	–	–
	ATR-101 (Lipid metabolism)	ABCA1/ ABCG1	Inhibition of cholesterol efflux	[73]	Human lung cancer tissue	–	–
	Olaparib, Niraparib, Talazoparib (Glucose metabolism; lipid metabolism)	SREBP PARP	Switch from glycolysis to lipid metabolism, metabolic reprogramming	[74]	BRCA1-associated TNBC	–	–

Similar to VEGF, the angiopoietins, like Ang2, which is a regulator of vessel wall integrity, and their tyrosine kinase receptors (Tie2/Tek) have been shown to play an essential role in angiogenesis [60, 64, 65, 75]. Various studies have shown that TIE2-expressing macrophages can promote tumor angiogenesis in human and mice, as seen in a mouse model of breast cancer [59, 61, 75]. It was also found that administering an antiangiopoietin mAb can effectively improve PFS in patients with ovarian cancer [59]. Blocking the interaction between Ang2 and Tie2 by Ang2 CovX-Bodies inhibits TME infiltration, which is beneficial for tumor control [23]. Furthermore, VEGF and Ang2 play complementary and coordinated roles in different stages of angiogenesis [57]. Dual Inhibition of Ang2 and VEGF through combination therapy or bispecific antibodies, such as the use of Ang2 inhibitors in combination with bevacizumab or aflibercept, has been more effective in TME exclusion, and antiangiogenic functions since upregulation of Ang2 can cause resistance to anti-VEGF therapy [23, 57, 65]. Also, dual inhibition of Ang-2/VEGF with a bispecific anti-Ang2/VEGFA antibody (CrossMab, A2V) prolongs the survival of patients and animal models of glioblastoma and sarcoma [23, 57, 65]. Studies have shown that A2V promotes vascular normalization and skews the M2-like protumor phenotype toward the M1-like proinflammatory phenotype in glioblastoma. Another example is tetravalent anti-VEGFA and anti-Ang2 bispecific antibody (TAvi6), which has been shown to abolish angiogenesis and improve antitumor efficacy [52]. Therefore, angiopoietins and Tie2 signaling have been proposed as targets for antitumor treatment because of their ability to inhibit tumors by blocking angiogenic signals and the immunosuppressive functions of TAMs [59].

Furthermore, Tie2 is upregulated in TAMs by CSF1, which indicates a link between CSF1, Tie2 + macrophages, and angiogenic switch stimulation [76]. It is well established that CSF-1 is necessary for macrophage differentiation and plays an essential role in developing the high-density blood vessel networks in the murine model through VEGF production [59, 77]. In this model, macrophage-produced WNT7b stimulates the production of VEGF in vascular ECs, resulting in an angiogenic switch [77]. CSF-1R inhibitors have been developed to inhibit tumor progression by suppressing macrophage differentiation toward the M2 phenotype and macrophage-related angiogenesis. [59].

In addition, various transcription factors and related signal transduction pathways are also involved in the proangiogenic processes. Hypoxia-induced factors (HIFs), for instance, mediate the metabolic switch of TAMs to oxidative phosphorylation, which is necessary for angiogenic function of TAMs [60]. Moreover, Joshi et al. showed that the PTEN/PI3K/AKT pathway could enhance hypoxia-induced HIF-1α and HIF-2α mediated induction of VEGF by macrophages stability of macrophages, thereby promoting angiogenesis in tumors of Lewis lung carcinoma [55]. Also, Dong et al. provided evidence that M2 macrophage-derived VEGF could simultaneously increase the expression of prostate cancer-associated transcript 6 (PCAT6) and VEGFR-2 through miR-4723-5p sponging, thereby enhancing tumor angiogenesis through the activation of VEGFR-2/Akt/mTOR signaling pathway [55, 78]. Moreover, trabectedin targets TAMs via the TRAIL receptor (ET743, Yondelis®) by activating the caspase 8 cascade. Trabectedin causes a selective TAM depletion and is accompanied by reduced angiogenesis [79]. Furthermore, WNT7b secreted by TAMs stimulates Wnt/βcatenin signaling in ECs and promotes angiogenic activity, which can be impaired by WNT7b deletion in myeloid cells [60]. WNT7b, a WNT family ligand, is produced in TAMs and is found to activate the VEGFA-mediated angiogenic switch by stimulating canonical WNT/β-catenin signaling [57, 61]. WNT7b was shown to be responsible for tumor growth and angiogenesis in a mouse model of breast cancer [61]. In contrast, it has been found that a Wnt ligand, WNT5a, which mainly activates non-canonical signaling pathways, can suppress vascular sprouting by upregulating soluble VEGFR1. These findings suggest that canonical and non-canonical Wnt signaling pathways may mediate opposing angiogenic responses. According to these findings, the inhibition of canonical Wnt signaling by inhibitors as well as agonist stimulation of non-canonical Wnt signaling may have promising antiangiogenetic strategies [57]. As mentioned above, another pathway involved in angiogenesis is STAT3 signaling, which promotes multidirectional interactions between TAMs, ECs, and tumor cells. STAT3 is constitutively activated in many cancers, and TAMs cause STAT3-dependent expression of proangiogenic molecules leading to stimulating STAT3 action in ECs, thereby promoting vascularization [80]. STAT3 is not only an essential molecule for the activation of tumor cells but a key mediator of angiogenesis, which increases the production of angiogenic factors like VEGF and basic fibroblast growth factor (bFGF), consequently leading to activation of STAT3 pathway in ECs and stimulation of vascularization [59]. Horiguchi et al. discovered that WP1066, a STAT3 inhibitor, suppresses the basal and hypoxia-induced expression of HIF-1α, HIF-2α and Bcl-2 and the secretion of VEGF, and also stimulates apoptosis [81]. Inhibition of mTOR by the addition of rapamycin, upregulation of TSC2 or blockade of STAT3 in monocytes/macrophages has been shown to promote the release of M1 cytokines and inhibit tumor growth, which was confirmed by reduced tumor angiogenesis. This finding suggests that mTOR could also regulate the ability of macrophages to induce angiogenesis [80]. It was also found that angiogenesis regulated by the TSC2–mTOR axis may be mediated by other growth factors, other than VEGF. STAT3 mediates the impacts of mTOR in macrophage-induced neoangiogenesis, potentially suggesting new insights into innate immune responses and new cancer therapy targets. These results suggest that inhibition of TSC2–mTOR–STAT3 in the innate immune response could be another novel and potent therapeutic avenue [80]. Upregulation of a negative regulator of mTOR, REDD1, has been shown in hypoxic TAMs, and inhibition of mTOR in TAMs has been shown to reduce excessive angiogenic response [82]. The underlying mechanism is the REDD1 depletion which can rescue mTOR activation. Conversely, it was found that upon inhibition of mTOR in macrophages in hepatocellular carcinoma (HCC), STAT3 decreased the secretion of both IL-10 and IL-12 and inhibited angiogenesis in vivo [80]. According to these findings, mTOR has a dual role in tumor angiogenesis. Therefore, mTOR-targeted strategies to reduce tumor angiogenesis should be warranted for further understanding of the underlying mechanisms and optimizing antitumor function [55].

Cellular metabolism was found to regulate TAM polarization, thereby influencing both protumoral and antitumoral responses [83]. Similar to T cells, TAMs also compete with their adjacent cells for glucose. Glycolytic activity in TAMs is predominantly associated with tumor regression. Hence, GAPDH activity is decreased in human M2 TAMs in contrast to M1 TAMs [84]. Furthermore, under hypoxic conditions, tumor-associated macrophages (TAMs) exhibit increased expression of the mTOR negative regulator REDD1, leading to reduced glycolytic activity. Intriguingly, TAMs lacking REDD1 outperform endothelial cells in utilizing glucose, thereby supporting tumor vessel normalization and hindering metastatic dissemination [85]. Conversely, when human blood monocytes are cultured with conditioned media from pancreatic ductal adenocarcinoma (PDAC) cell lines, it fosters the development of TAMs exhibiting elevated glycolytic activity and enhanced metastatic capabilities. The use of glycolysis inhibitors impeded this process, indicating that the metabolic nutrient modulated by cancer cells significantly influences the phenotype and function of TAMs [71].

Moreover, targeting tumor-derived metabolites, including lactate, adenosine, and glutamine, has been investigated in preclinical models due to the effects of these metabolites on macrophages and tumors [86]. Lactate produced by tumor cells has a critical function in signaling and TAM polarization. Lactate, for instance, a pivotal factor in driving macrophage M2 polarization to promote breast cancer progression, has been shown to activate ERK1/2 and STAT3 pathways in macrophages. Furthermore, it has been found that WithaD, a chemotherapeutic agent, effectively prevents macrophage M2-polarization by decreasing ERK/STAT3 pathway activation, suppressing breast cancer progression and tumor angiogenesis by eliminating lactate-induced M2 macrophage polarization [87]. Moreover, inhibiting the ERK/STAT3 axis with selumetinib or stattic, or the inhibition of Gpr132 has been shown to lower lactate-induced M2 polarization and demonstrate significant antitumor effects in preclinical murine models [87, 88].

Recent research has shown that lactate has the capacity to induce epigenetic alterations in M1 macrophages challenged by bacteria. This process, termed histone lactylation, triggers the upregulation of genes crucial for maintaining homeostasis. Given the elevated lactate levels within the TME, future investigations must delve into the significance of histone lactylation in the interplay between tumor cells and macrophages [89]. Similarly, a glutamine synthetase inhibitor, glufosinate, was reported to reduce metastasis by reprogramming TAMs into antitumor agents in highly metastatic mouse models, with the antitumor effect related to reduced angiogenesis and immunosuppression [86]. The glutamine metabolism within TAMs is associated with a protumoral characteristic facilitated by the generation of α-ketoglutarate. This compound aids in prompting fatty acid oxidation (FAO) and epigenetically activating M2 genes [90]. Furthermore, glutamine-synthetase (GS) expression and activity are increased in the M2 phenotype, becoming particularly significant during starvation conditions. Blocking GS activity in macrophages triggers a transition from an M2 to an M1 phenotype, ultimately impeding metastatic dissemination [74]. In summary, these investigations delineate a unique metabolic signature differentiating M1 from M2, resembling the metabolic features of cancer cells. Consequently, targeting precise metabolic pathways could offer dual benefits: directly impeding tumor progression and indirectly inducing a shift toward an M1 antitumoral phenotype.

## TAMs as a target for hindering tumor invasion

The ECM provides both the biochemical and essential structural support for tumor growth; however, it is also an important barrier to the spread of tumor cells. Therefore, basement membrane degradation is considered as a vital process for the escape of tumor cells from the local environment [52].TAMs promote invasion and metastasis of cancer cells in various ways, such as secretion of vital proteinases (MMPs, cathepsin, etc.) and many other proteases disintegrating ECM [52, 91, 92]. MMPs, including collagenase (MMP1), gelatinase A (MMP2), stromelysin (MMP3), matrilysin (MMP7), gelatinase B (MMP9), are a group of zinc-dependent proteases involved in matrix degradation [93]. TAMs also secrete growth factors, including PDGF, EGF, TGF-β, and hepatocyte growth factors (HGF) to induce the proliferation and invasion of tumor cells. Data obtained from clinical specimen analysis showed that TAMs were enriched at the site of the high incidence of the epithelial-mesenchymal transition (EMT), giving tumor cells a migratory and invasive phenotype [52, 94, 95].

Invasive tumor cells and macrophages have been found to co-migrate in primary mammary tumors of mice and rats only in response to EGF and CSF-1 [96]; thus, blockade of either EGF receptor or CSF-1 receptor signaling blocks migration of both cell types in vivo [97]. EGF is a proinvasive factor that activates the EGFR-ERK pathway, leading to the induction of EMT of cancer cells, cancer cell invasion and metastasis [98, 99]. A paracrine EGFR/CSF-1R interaction exists between TAMs and tumor cells in which CSF-1 stimulates macrophages to release EGF, leading to tumor cell proliferation and migration. EGF also stimulates CSF-1 secretion by cancer cells, forming a positive feedback loop between macrophages and tumor cells [93]. A key observation is that stimulating macrophages by CSF-1 and tumor cells by EGF induce podosomes and invadopodia formation, respectively, which can mediate basement membrane and tissue invasion of tumor cells. Thus, ECM remodeling activity and invasive properties are exaggerated by each type of cell simultaneously, leading to the increased migratory activity. These results showed the involvement of macrophages in the induction of tumor cell migration and invasion in the primary tumor [97]. Both preclinical and clinical studies have shown that targeting the CSF-1/CSF-1R axis can be a promising therapeutic strategy, as using antagonists and mAbs for CSF-1R have great potential to improve the outcome of patients with advanced cancer [60].

With increasing evidence supporting the crosstalk between TAMs with EMT in tumor metastasis, targeting the signaling pathways and other stimulators in this process is an important option for invasive cancer treatment. The signaling cascades induced by TGF-β, for example, play an important role in this crosstalk making the TGF-β-induced signaling pathways as potential therapeutic targets. For example, CX-4945, a selective and potent inhibitor of protein kinase CK2, inhibited the EMT-mediated invasion and migration of A549 human lung cancer cells through blocking the TGF-β1 signaling cascade. Moreover, A549 cell migration and invasion was suppressed through inhibiting the TGF-β1-induced EMT by Ginsenoside 20 (R)-Rg3 which is an active component of ginseng [100]. TGF-β was found to upregulate HIF-1α, which increases the tribbles pseudokinase 3 (TRIB3) expression in CRC cells, leading to the activation of β-catenin/Wnt signaling pathway, thereby promoting the invasive capability of CRC cells. Moreover, Fan et al. demonstrated that CD68 + TAMs showed the M2 phenotype with a higher TGF-β1 expression which induced the EMT process and promoted the invasiveness of HCC, while administration of TGF-β1-neutralizing antibody attenuated the TGF-β1-induced EMT, invasion, and migration of HCC [101]. Also, Okamoto et al. showed that TAM-derived growth differentiation factor 15 (GDF15) increased phosphorylation levels of TGF-βRII and invasion of esophageal squamous-cell carcinomas (ESCC), while LY2109761, a TGF-βRI/II inhibitor, suppressed GDF15-dependent enhancement of ESCC invasion [102]. Accordingly, TGF-β and GDF15, a member of the TGF‐β superfamily, play a key role in tumor invasion and EMT, which is considered an essential mechanism in TGF-β-induced tumor invasion.

Additionally, a proinflammatory cytokine secreted by TAMs in the TME, i.e., TNF-α, is known to support cancer cell invasion [55]. Co-culture of ovarian or breast cancer cells and TAMs can trigger the activation of nuclear factor kappa-B (NF-κB) and c-Jun N-terminal kinases (JNK) pathways in a TNF-α-dependent manner, which leads to increased invasion of tumor cells [103]. In addition, Cho et al. showed that TPCK, a TNF-α inhibitor, could reduce ovarian cancer invasiveness, suggesting that TAM-derived TNF-α promoting cancer invasion is a common event in tumor progression [104]. A study by Singh et al. supported these findings and found that macrophage-derived TNF-α could induce TGF-β1 secretion in breast cancer cells leading to DNA damage by activating a survival pathway to deregulate DNA damage and reactive sxygen species (ROS), subsequently causing EMT enhancement by increasing the expression of CREB phosphorylation and vimentin, while the neutralization of TNF-α by GolgiPlug (555,029) showed to abrogate invasion and migration of breast cancer cells [105]. TNF-α has also been shown to induce EMT and promote renal cell carcinoma (RCC) tumorigenesis and invasiveness by suppressing E-cadherin, upregulating vimentin, and increasing MMP9 expression [106]. In a study conducted by Watanabe et al., it was shown that exogenous recombinant TNF-α could stimulate the secretion of IL-8 in oral squamous cell carcinoma cells, leading to increased tumor invasion with the degradation of ECM by increasing MMP2/7/9 release [107]. The IL-8 signaling pathway also contributes to the proliferation, invasion, and stemness of glioblastoma cells [60]. For example, blocking the upstream signal of the IL-8 pathway, calcineurin, by cyclosporine was found to inhibit tumor growth and invasion through IL-8 depletion [108]. A study by Fitzgerald et al. demonstrated that increased levels of IL-6 and IL-8 also lead to the invasion of RCC cells [109]. Moreover, IL-6-induced RCC proliferation is mediated by enhanced DNA binding activity of STAT3 and, to a lesser extent, STAT1 [81]. In conclusion, recombinant and TAM-derived TNF-α plays a promoting role in the invasion of tumor cells through various signaling pathways [55].

Increasing in vitro and in vivo evidence indicate that PI3K signaling is critical for driving M2 polarization and tumor cell invasion induced by TAMs. [77]. For example, the HB-EGF-EGFR-PI3K/Akt downstream signaling pathways have been found to play a key role in TAM-induced cell motility and invasion of myxoid liposarcoma (MLS) [110]. PI3K activation was also shown to enhance invasion by regulating the expression of various MMPs, like MMP2 in mouse mammary epithelial cells [111] or MMP9 in HT1080 cells [112, 113]. Fortunately, two PI3K inhibitors, namely copanlisib and duvelisib, have received approval for medical use [114, 115]. Copanlisib has been approved as a pan-PI3K inhibitor for adult patients with follicular B cell non-Hodgkin’s lymphoma who have relapsed after at least two prior lines of systemic therapies [77]. Further, PI3Kγ, abundantly expressed on macrophages, can inhibit the activation of NF-κB and subsequently promote immune suppression during tumor growth [23]. Inhibition or deficiency of PI3Kγ ameliorates the immunosuppressive status by reprogramming macrophages and inhibiting metastasis and invasion of tumor cells in various animal models, such as pancreatic ductal adenocarcinoma and breast cancer [116–119].

## TAMs as a target to inhibit tumor cell intravasation, circulation and extravasation

Tumor metastasis is a major contributor to the mortality of cancer patients. Numerous studies have shown that TAMs function as a prominent promoter of metastasis in the TME, coordinating all steps in the tumor metastasis cascade. TAMs are involved in the processes of metastasis by producing growth factors, chemokines, proteases and different types of immune checkpoint inhibitory proteins in T cells [120]. Recently, targeting TAMs has attracted extensive attention as a therapeutic strategy to prevent tumor progression and metastasis.

Intravasation is a major stage in the metastasis, in which cancer cells invade a blood vessel. Studies have shown that macrophages enhance the ability of cancer cells to invade the vessels. Using imaging techniques, macrophages are found on the periphery of the tumor and in blood vessels helping cancer cells enter the bloodstream [97]. Cancer cells secrete CSF1, thereby stimulating macrophages to produce EGF which in turn triggers the migration of cancer cells [121]. EGF and CSF1 induce the formation of invadopodia in tumor cells and podosomes in TAMs, structures that break the ECM and facilitate intravasation [97]. Therefore, the inhibition of the CSF-1 or EGF signaling pathway may have promise to disrupt the migration of both cell types and reduce the number of circulating tumor cells (CTCs) [122].

Generally, two subsets of TAMs support the inside of cancer cells: migratory and perivascular macrophages [123, 124]. Migratory macrophages guide cancer cells to blood vessels, while perivascular macrophages help them enter the bloodstream. Motile TAMs are newly arrived monocytes, recruited through CCR2 signaling, which then differentiate into perivascular macrophages. This process is regulated by CXCL12 and CXCR4. Cancer cells induce TGF-β-dependent stimulation of CXCR4 in monocytes, while CXCL12 expressed by perivascular fibroblasts recruits these migratory TAMs to the blood vessels and entrains the motile cancer cells. Once located on the blood vessel, migrating TAMs differentiate into perivascular Tie2-expressing macrophages that mediate cancer cell intravasation by expressing VEGFA and causing vascular leakage [125]. Therefore, inhibition of CXCR4/CXCL12 or Tie2 kinase may serve as a target to prevent tumor intravasation. The inhibition of Tie2 kinase or blocking the Tie2 ligand Ang2 have been reported to prevent the intravasation and metastasis in the PyMT mammary tumor model [126]. A study showed that treatment with AMD3100, a C-X-C chemokine receptor type 4 (CXCR4) antagonist, led to a reduction in perivascular macrophages [127].

After penetrating the blood vessels, tumor cells must prepare for survival and exit from the circulation. Clots enclosed around tumor cells prevent elimination by natural killer cells in a tissue factor-dependent manner in the general circulation and capillaries [128]. In a study, it was shown that by disrupting the function of macrophages, the survival of tumor cells in the pulmonary capillaries was reduced and lung metastasis was not observed despite clot formation, indicating the essential role of macrophages in this aspect [129]. A recent study in breast cancer cells reported that recruited macrophages trigger the PI3K/Akt survival signaling pathway by engaging vascular cell adhesion molecule-1 (VCAM-1) via α4 integrin [130, 131]. Activation of the PI3K/Akt survival pathway subsequently rescued cancer cells from proapoptotic cytokines such as TNF-related apoptosis inducer [130]. In fact, the reason why many tumor cells survive is due to chemokines or cytokines secreted directly from macrophages [129].

When located in the capillaries of target organs, tumor cells try to stick and extrude from the walls of the vessels with the help of macrophages. Intimate contacts between tumor cells and macrophages during extravasation were observed and quantitatively analyzed in a lung imaging system. Of note, it has been implied that the rate of extravasation and metastasis is significantly reduced following the loss of macrophages [132].

Macrophages play a major role in tumor cell extravasation, by establishing the premetastatic niche at distant metastatic sites [132]. CCL2-CCR2 signaling pathway promotes the early recruitment of inflammatory monocytes to the premetastatic niche, where the recruited monocytes develop into metastasis-associated macrophages (MAMs). MAM-derived VEGFA promotes the extravasation and seeding of tumor cells [133]. Moreover, CCL2-CCR2 signaling also activate the CCL3-CCR1 (receptor of CCL3) signaling in MAMs, which support MAM accumulation at the metastatic site. This process promoted the extravasation and seeding of the breast cancer cells in several mouse models of breast cancer metastasis (13). In addition, TAM production of IL-1β, induced by CCL2, resulted in systemic inflammatory cascades leading to the neutrophil-mediated promotion of mammary tumor metastasis in mice [134].

Interestingly, platelets protect tumor cells from being detected by cytotoxic immune cells via being accumulated with CTCs. Platelets accompany tumor cells in the circulation to the site of extravasation, where they help tumor cells exit the circulation into the secondary organs. Schumacher et al. reported that platelets increase the extravasation of tumor cells and metastatic seeds through ATP-dependent activation of the endothelial P2Y2 receptor, which opens the vascular barrier [135]. However, using an imaging system, Qian et al. showed that tumor cells interacting with macrophages are associated with a higher percentage of extravasation, while the reduction of macrophages using L-clodronate, strongly reduced the number of tumor cells undergoing extravasation [132].

## Recent TAM-related Therapeutic targets in different cancers

### Gastric *cancer*

Gastric cancer is the third leading cause of cancer deaths worldwide [136]. Although various treatments, including surgical resection, radiation therapy, chemotherapy are used for the treatment of this daunting malignancy, the mortality rate of gastric cancer remains high (103). Therefore, traditional treatments need to be improved, and new treatment strategies should be developed [137]. Since TAMs are frequently observed in gastric tumors, and they interact with tumor cells in different ways, they can be used as novel therapeutic targets for the treatment of patients with gastric cancer [137, 138]. To date, several key pathways like chemokine-chemokine receptor signaling, receptor tyrosine kinase (RTK) signaling, metabolic signaling, and exosomal signaling have been highlighted in discovering the biological functions of TAMs. Promising aspects of targeting each signaling in gastric cancer are described below [139].

As mentioned above, TAMs could promote cancer cells' growth, invasion, and metastasis by secreting several chemokines and cytokines [140]. Targeting chemokine–chemokine receptor networks have been assessed in several preclinical models and clinical trials, particularly CCL2-CCR2, CCL5-CCR5, and CXCL12-CXCR4 axes [139, 141]. The development of CCR2 inhibitors has brought positive results in cancer treatment [139, 142], and multiple CCL2-neutralizing antibodies are being tested in clinical trials [142]. Overexpression of CCL2 induces angiogenesis and tumorigenesis of gastric cancer cells in nude mice through macrophage recruitment. A high level of CCL2 has also been found in gastric cancer patients, and it was correlated with lymph node metastasis and reduced overall survival (OS) [141]. The overexpression of CCL2 derived from HER2-positive gastric cancer cells caused a decrease in M1 TAMs density and recruited the M2-like phenotype of TAMs, thereby promoting resistance to trastuzumab. Using CD40 × HER2 bispecific antibody showed great antitumor efficacy, to overcome trastuzumab resistance in HER2-positive gastric cancers [143]. Another agent is Sophoridine which has been reported to suppress M2 polarization and increase M1 polarization through TLR4/IRF3 pathway and inhibit TAMs infiltration by downregulating CCR2 expression in gastric cancer TME, thereby improving the cytotoxicity function of CD8 + T cells [137, 144]. Zhuang et al. showed that sophoridine upregulated IL-12α and TNFα while downregulated IL-10 and CD206 through the TLR4/IRF3 signaling in gastric cancer TME, enhancing TAMs repolarization toward the M1-type in gastric cancer [137, 145]. Sophoridine also induces G2/M cell cycle arrest by inhibiting double-stranded DNA break repair and improving the effectiveness of cisplatin in gastric cancer cells. Thus, sophoridine may have promises as a novel therapeutic for gastric cancer treatment [145]. These findings provide preclinical evidence supporting CCR2-CCL2 as a promising target for human gastric cancer treatment, especially when combined with traditional treatment strategies [146]. Targeted blockade of CCL5 is also effective in several malignancies. Treatment with maraviroc in gastric cancer, for example, effectively inhibited peritoneal dissemination, decreased tumor burden, and prolonged the survival rate [146]. CCL5 was overexpressed in cancerous tissues and serum of gastric cancer patients, and its level was closely related to the differentiation, growth, pathological stage, invasion, and metastasis of gastric cancer. CCL5 expression was also positively correlated with TAM surface marker CD68, showing the involvement of TAMs in the development and metastasis of gastric cancer through secreting CCL5. Therefore, CCL5 may be a potential target for future treatment strategies for gastric cancer patients [140]. Since CXCL12-CXCR4 is the most overexpressed pathway in various cancers, several small molecule drugs and peptide inhibitors targeting CXCR4 have also been developed [146]. CXCR4 and its ligand, CXCL12, are immunohistochemically overexpressed in gastric cancer compared with normal gastric tissue and are associated with cancer cell survival, proliferation, angiogenesis, and migration. Recently, several preclinical studies in various tumor types, including gastric cancer, have reported encouraging antitumor effects from this biological class [141]. An interesting therapeutic strategy is to combine CXCL12-CXCR4 inhibitors with other targeted agents that block the same pathways, such as MEK and mTOR inhibitors, or with other chemokine inhibitors [141]. According to these findings, disrupting the CXCL12-CXCR4 pathway represents a successful strategy for anticancer drug development [146].

Administration of cytokine (IL-1, IL-6, IL-18, TNF) antagonists is the other therapy for gastric cancer patients. IL-1β expression is notably correlated with clinical and pathological features of the patients. Elevated levels of IL-1β are correlated with advanced metastatic disease in human xenograft models [141]. Moreover, IL-1R pathway might be associated with intrinsic aggressiveness of gastric cancer cells [144]. The administration of IL-1 receptor antagonist (anakinra) resulted in reduced tumor growth, reduced angiogenesis, and reduced metastases in murine xenograft models [141]. The good absorbance of this agent in humans and its safety make it a candidate to be tested alone or in combination with chemotherapy in the patients [141, 144]. IL-8 enhanced EC proliferation and migration as well as increased rate of EMT. It also modulates ECM remodeling by increasing the expression of MMPs, such as MMP2 and MMP9. Repertaxin, an inhibitor of IL-8 receptors, CXCR1 and CXCR2, has been reported to reduce tumor proliferation in gastric cancer cell line MKN45. Meanwhile, direct blockade of IL-8 has also received much attention, especially because findings suggest that high serum levels of IL-8 is correlated with poor prognosis in several tumors, including gastric cancer. Gastric cancer cell lines also express high levels of the IL-6 receptor, the activation of which leads to STAT3 activation and VEGF production. Napabucasin, an oral inhibitor of cancer stem cells acting by blocking STAT3 signaling, is being tested in various gastrointestinal tumors, either alone or in combination with standard chemotherapy. An interesting strategy in gastric cancer is to investigate the efficacy and safety of napabucasin in combination with an anti-IL-6 antagonist [141].

The third strategy is to administrate the agents which target tyrosine kinases. Since the CSF-1/CSF-1R axis is activated in multiple malignancies and is the most important pathway associated with TAM recruitment and proliferation, developing agents to block this pathway has attracted increasing research attention [141, 142]. Currently, CSF-1/CSF-1R antibodies and CSF-1R kinase inhibitors have been successfully developed for therapeutic regulation of macrophages. In the context of gastric cancer, multiple clinical trials involving patients are underway to investigate the clinical efficacies of CSF-1R inhibitors in cancer treatment [141]. However, the blockade of CSF-1R alone usually achieves marginal therapeutic benefits and, at most, results in delayed tumor growth [146].

The other possible therapeutic strategy is to use cell signaling inhibitors. The inhibition of transcription factor NF-κB is a therapeutic modality to target multiple cytokines/inflammatory mediators. Multiple drugs are available to modulate the NF-κB pathway, thereby reducing the expression of IL-8 and other proinflammatory cytokines. Many of these drugs, including resveratrol, apigenin, anthocyanin, and RK-1–123, target NF-κB indirectly by decreasing ROS production. Resveratrol has received significant attention as a potential treatment/adjunct therapy for gastric cancer patients since it is a member of the polyphenol flavonoids class of antioxidants produced by a restricted number of plants [141]. Moreover, various studies showed that targeting the PI3K-γ signaling pathway in TAMs may provide a novel potential therapeutic approach to improve the long-term survival of the patients. Methionine enkephalin (MENK), an endogenous opioid Penta-peptide, possesses immunotherapeutic activity and promotes the polarization of M2-type TAMs to M1-type [147]. Several studies have shown that MENK could exert its antitumor function by stimulating TAM polarization from the M2 to M1 phenotype in gastric cancer [137]. MENK reduced the expression of PI3K, p-AKT, and mTOR, suggesting that MENK could inhibit PI3K/AKT/mTOR signaling in gastric cancer [148]. Cholecystokinin (CCK)-B receptor signaling is another essential pathway in stimulating cancer growth. Strategies to disrupt the gastrin interaction have the potential to inhibit cancer cell growth since exogenous administration or endogenous production of gastrin from cancer cells can activate the CCK-BR receptor, leading to tumor proliferation. Polyantibody stimulator (PAS) vaccine that targets gastrin reduces cancer proliferation when administered as monotherapy. However, the tumor inhibitory effect of this vaccine was significantly improved when administrated with PD-1 antibody, since PAS increased the number of CD8 + T cells and decreased the number of M2-type macrophages, making the TME more sensitive to other treatments. According to these findings, the CCK-BR receptor can be a potential target for human gastric cancer therapy [149].

Using immune checkpoint inhibitors, including anti-PD-1/PD-L1 and anti-CTLA4, provides another therapeutic approach. The PD-1/PD-L1 signaling pathway has become the hot spot of current immunotherapy for gastric cancer, and targeting M2-type TAMs is a practical approach to modulate the activity of anti-PD-1/PD-L1 agents. According to a study by Wang et al., TAMs from gastric cancer patients shared markedly increased PD-1 levels, leading to tumor progression by impairing the antitumor functions of CD8 + T cells [137]. Two immune checkpoint inhibitors, pembrolizumab, and nivolumab, both of which are anti-PD-1 mAbs, have been approved in many countries for advanced/metastatic gastric cancer [139, 141, 150]. Moreover, Lenvatinib, a multi-kinase inhibitor, decreased TAMs levels, enhanced interferon signaling activation, and improved the antitumor function of PD-1 inhibitors. Lenvatinib combined with pembrolizumab showed promising antitumor roles in gastric cancer [137].

### Breast *cancer*

Regarding the role of TAMs in the development and metastasis of breast tumors, studies have found that these cells can promote angiogenesis and ECM remodeling and also could enable tumor cells to evade the immunity [87]. Since it has been proven that TAMs can be indicative of poor prognosis and chemoresistance due to their tumor-promoting and immunosuppressive functions, they have become potential targets for therapeutic intervention in various cancers, including breast cancer [61]. Selective inhibition of VEGFR2, for instance, caused decreased macrophage infiltration and reduced angiogenesis in breast cancer models [79]. Also, CSF-1/CSF-1R inhibitors, including kinase inhibitors and antibodies, can attenuate angiogenesis, metastasis, and proliferation of tumor cells and reprogram cancer cell growth in breast cancer models [77, 151]. A CSF-1R antibody, emactuzumab (RG7155), blocks dimerization and activation of CSF-1R and results in decreased infiltration of CD163 + TAMs in different solid tumors [152, 153]. Treatment of mammary tumor-bearing mice with PLX3397 (Pexidartinib), a CSF-1R tyrosine kinase inhibitor, significantly reduced the number of TAMs and resulted in delayed tumor growth stimulated by an increase in CD8 + T cells and a decrease in CD4 + T cells [60]. Moreover, deletion of CSF-1 leads to reduced breast cancer incidence, slower tumor progression, and decreased metastasis [153]. Ramesh et al. focused on inhibiting the CSF-1R and MAPK signaling, a significant axis in the activation and proliferation of macrophages, by developing a lipid nanoparticle formulation filled with a dual CSF-1 and MAPK kinase inhibitor. This inhibitor led to repolarization of M2 macrophages to an M1 phenotype in the TME, showing suppressed tumor growth and decreased toxicity in a highly aggressive breast cancer model [154]. Also, agonistic anti-CD40 antibodies not only exert tumor suppressive effects in various cancer mouse models, but their combination with anti-CSF-1R antibodies leads to profound TAM reprogramming before their depletion. These reprogrammed TAMs create a proinflammatory milieu showing effective T cell responses even in tumors unresponsive to immune checkpoint inhibitors. CP-870,893, APX005M, and RO7009789 are agonistic anti-CD40 antibodies being tested in clinical trials for different types of cancers. In general, CP-870,893 well tolerated by patients, and CP-870,893 caused an objective response and antitumor activity [30, 155].

TAMs, have been found to induce resistance to tamoxifen in breast cancer patients [156, 157]. Following neoadjuvant chemotherapy (NAC), the accumulation of tumor-associated macrophages in breast cancer cells has been identified in patients and animal models. In a study on 311 Swedish breast cancer patients, higher levels of tumor-infiltrating CD45 + CD11b + CD14 + macrophages were found in women who received NAC (paclitaxel and fluorouracil-doxorubicin-cyclophosphamide) than in those who underwent only surgery [156]. For example, administration of paclitaxel can increase macrophage chemotactic factors (MCFs), such as IL-34, CCL8, CSF-1, and CSF-1R [153]. Upregulation of CSF-1 in tumor cells following exposure to paclitaxel was correlated with high TAM infiltration*,* and the recruited TAMs suppressed paclitaxel-induced mitotic arrest in breast tumor cells. Therefore, blocking the CSF1-CSF1R axis along with chemotherapy leads to enhanced paclitaxel efficacy and prolonged survival of the mice, accompanied by improved cytotoxic T lymphocyte (CTL) and decreased blood vessel density by suppressing VEGF expression [152, 153, 158]. Numerous research on mouse models confirmed the effectiveness of treatment based on a combination of chemotherapy drugs and TAMs inhibitors in tumors [156]. Combined chemotherapy treatment (methotrexate, 5-fluorouracil, CMF— cyclophosphamide) and anti-CSF-1 Fab administration [murinized, polyethylene glycol-linked antigen-binding fragment (Fab) against mouse (host) CSF-1] in mice bearing chemoresistant MCF-7 breast cancer xenograft suppressed tumor development, reduced angiogenesis, downregulated expression of chemoresistance-related genes, and increased the survival rates [156]. TAMs also inhibited the efficacy of other chemotherapeutic drugs, such as doxorubicin, gemcitabine, etoposide, and CMF regimen (cyclophosphamide, methotrexate, 5-fluorouracil) [152]. Moreover, TAM-derived cathepsins especially cathepsin S and cathepsin B might be one of the mechanisms contributing to TAM-mediated tumor chemoresistance in breast cancer, protecting murine mammary tumor cells from paclitaxel-, doxorubicin-, etoposide-induced cell death [61]. Anticathepsin D antibody has also been identified to inhibit TAM recruitment by decreasing TGF-ß levels and thus inhibiting tumor growth in triple-negative patients. Administration of PTX in combination with cathepsin deletion by cysteine cathepsin inhibitor (PM-OEt significantly improved the therapeutic effectiveness of PTX, suppressed metastatic burden and tumor growth, and improved late-stage survival [156]. TAM-derived IL-10 is the other factor with chemoprotective effects. IL-10-mediated drug resistance is associated with the upregulation of STAT3 signaling and antiapoptotic protein Bcl-2 in tumor cells. Antibody-mediated inhibition of IL-10 reversed paclitaxel resistance in ex vivo co-culture studies. This was already shown for TMP195, a selective class IIa histone deacetylase (HDACIIa) inhibitor that reprograms TAMs toward M1-like phenotypes, decreases tumor burden, and enhances antitumor effects in MMTV-PyMT mice, especially when combined with paclitaxel [156, 159]. TMP195 enhanced its tumoricidal effect in a mouse breast cancer model when combined with chemotherapy or checkpoint blockade [159]. Su et al. proved that after administration of anti-HER2 antibodies combined with PD-L1 and indoleamine 2,3-dioxygenase (IDO) inhibitors, the efficacy of antitumor immunity and anti-HER2 treatment were improved in mouse models. As an example, administration of the CSF-1R inhibitors in combination with checkpoint blockade-based immunotherapy causes a delay in tumor progression in mouse models of various cancers, including breast cancer [79]. Also, intraperitoneal injection of TMP195 improved the antitumoral effects of TAMs, chemotherapy and PD-1 treatment in a luminal B-type breast cancer model [52, 152]. It also showed synergized effects with PD-1 antibody to decrease tumor burden and metastasis in an autochthonous mouse model of breast cancer [79]. These findings suggest that TAM-targeted therapy could be a potential strategy to overcome chemoresistance and enhance the effectiveness of checkpoint blockade-based immunotherapy in breast cancer [79, 152].

Myeloid cells, including macrophages, express signal regulatory protein alpha (SIRPα), a receptor that recognizes CD47 and, when binds to it, provides a down-regulatory signal preventing the phagocytosis of tumor cells. In addition to suppressing primary tumor progression and reducing metastasis, anti-CD47 treatment can synergize with immune checkpoint inhibitors to augment anti-neoplastic effects in some preclinical models [160]. Several drugs targeting CD47-SIRPα pathway are in early clinical development, including the ongoing phase II study of BTH1677 with pembrolizumab in patients with metastatic triple-negative breast cancer, in which more complete assessments demonstrated depolarization from M2-like TAMs to M1 phenotype [153].

Moreover, CCL2 promotes macrophage accumulation in the TME, functioning as a potent chemoattractant for monocytes/macrophages [155, 157]; thus blocking monocyte recruitment can be achieved by targeting the CCL2-CCR2 signaling axis [30, 92, 158]. Antibody-mediated CCL2 blockade inhibited monocyte recruitment to primary breast tumors, which led to tumor growth suppression and improved patient survival [30, 158]. Although anti-CCL2 mAb significantly reduced TAMs in the TME during treatment, withdrawal of the antibody leads to accelerated metastasis of breast cancer cells due to the rapid rebound of monocyte recruitment [23, 28, 30, 38, 155, 158, 161]. The mentioned effects are probably mediated through increased CCL2 production by tumor cells in response to anti-CCL2 therapy, which dramatically increases the availability of CCL2 after treatment cessation [158]. The mean serum levels of chemokine CCL22 were significantly higher in breast cancer patients than in healthy individuals. It was also shown that the CCL22 serum levels increases with advancing tumor stages, indicating that the elevated levels of CCL22 may contribute to breast cancer progression and development. CCL22 production by macrophages is increased by Th2-type cytokines, like IL-4 and IL-5, and decreased by Th1-type cytokines, such as IFN-γ. Higher levels of Th2-type cytokines have been found in the TME and peripheral blood of breast cancer patients [162, 163]. It was also found that the combined function of IFN-γ, TNF-α, and IL-1β will stimulate CCL22 secretion by tumor cells [163]. Also, a feedback loop between TAM-released CCL2 and activated PI3K/Akt/mTOR pathway in tumor cells is a possible mechanism for endocrine resistance in breast cancer patients [164]. Estradiol was reported to enhance macrophage influx and angiogenesis by increasing the release of CCL2, CCL5, and EGF in estrogen-positive breast cancers [153]. Interestingly, anti-CCL2 antibodies have shown to improve treatment efficacy when administered in combination with chemotherapy [38].

Due to the fact that activation of toll-like receptors (TLRs) expressed on macrophages results in M1 polarization and robust production of proinflammatory cytokines consisting TNF-α, IFN-γ, and IL-12 through NF-κB pathway, the ability of TLR agonists to reprogram TAM functions in destroying tumor cells have been evaluated in various mouse models. For example, the FDA-approved TLR7 agonist, Imiquimod, enhanced T cell recruitment and infiltration into tumors, inhibited breast cancer growth in mouse models, and synergized with immunotherapy to achieve complete tumor regression [30, 151]. Recent studies have reported that monophosphoryl lipid A (MPLA), a TLR4 agonist, and IFNγ, both FDA-approved biological agents, reprogrammed TAMs toward M1-phenotype by inducing type I interferon signaling pathway and activation of cytotoxic T cells through IL-12 and TNFα secreted by macrophages, leading to inhibited metastasis and decreased tumor growth in a breast cancer mouse model [79].

Some signal transduction pathways, e.g., NF-κβ, STAT3, and STAT6, ERK1/2, HIF-1α, etc., are involved in M2 macrophage polarization [87]. Therefore, inhibiting these pathways may have therapeutic potential in preventing tumor progression [153].

The activation of macrophages in response to M1 stimuli, like TLR ligands, IL-1β, or TNF-α, as well as the transcription of various tumor-promoting genes, including VEGF, IL-6, and COX2, is primarily regulated by NF-κB [81]. Since NF-κB is one of the crucial cytokines involved in the crosstalk between TAMs and tumor cell EMT, targeting NF-κB signaling has become an intensely studied strategy for treating TAM-related tumor metastasis. Zoledronic acid, for example, is a bisphosphonate used for treating bone metastasis in breast cancer patients which resulted in reversed EMT in triple-negative breast cancer cells by inactivating the NF-κB pathway [93]. Liposomal zoledronic acid also led to a decrease in TAMs and M2 marker CD206 and suppressed expression of CD31, followed by reduced angiogenesis and breast tumor growth in triple-negative breast cancer patients [153].

CXCL12–CXCR4 and Ang2–Tie2 are other pathways involved in macrophage recruitment. Radiation, chemotherapy, and vascular disruption increased CXCL12 expression and promoted CXCR4-dependent macrophage repopulation as well as resistance to breast cancer treatment. CXCL12 plays its role by recruiting Tie2 + macrophages, a population strongly associated with vasculature and tumor angiogenesis. Ang2 neutralization improved responses to VEGFA blockade, and Tie2 inhibition blocked chemotherapy-induced Tie2 + TAM recruitment in breast models and reduced metastasis [161]. Indeed, CXCR-4 targeting significantly reduced total tumor burden and metastases in different preclinical models [165]. Some products released in TME by TAMs can also inhibit TAM-mediated tumor growth, among which the CXCL1 inhibitor, XIAOPI formulation released from TAMs, was able to prevent premetastatic niche formation and cell proliferation, thus inhibiting breast cancer metastasis. A specific inhibitor of heat-shock protein 32 (HSP32) and heme oxygenase-1 (HO-1), zinc protoporphyrin IX (ZnPPIX), has also shown potential effects against breast cancer growth through HO-1 inhibition in vitro and in vivo. Moreover, inhibition of HO-1 can stimulates tumor-associated immune response through activating TAMs alternative proliferation, suggesting HO-1 as a potential target of breast cancer treatment [153].

### Ovarian *cancer*

Increased expression of CSF-1 and CSF-1R has been associated with poor prognosis in clinical studies of epithelial ovarian cancer (EOC). It has been found that proliferation, recruitment, differentiation from a tumoricidal to a tumor-promoting phenotype, and survival of macrophages depend on CSF-1/CSF-1R axis [60, 166–169]. CSF-1R, as a member of the tyrosine kinase receptor family, can induce its homodimerization upon binding to its ligands, CSF-1 or IL-34, and subsequently activate receptor signaling. Consequently, CSF-1R antagonists and mAbs can be used to prevent receptor dimerization and decrease macrophage survival [60]. Targeting CSF-1R repolarizes M2-like TAMs to the M1 phenotype, reduces the infiltration of macrophages into tumor tissue, and improves patients’ response to standard treatment in mouse ovarian tumor models and ovarian cancer patients [170]. However, anti-CSF-1R agents are mainly used combined with other compounds and molecules, like taxanes or anti-PD-1 antibodies [139, 169]. A selective CSF-1R kinase inhibitor named GW2580 significantly reduced ascitic fluid accumulation and infiltration of M2-type TAMs in a syngeneic mouse model of ovarian cancer. Furthermore, inhibition of CSF-1R partially overcomes anti-VEGF resistance, and disruption of CSF-1R leads to macrophage depletion, suggesting a direct role of CSF-1R in macrophage recruitment. The ongoing clinical trials targeting CSF-1R on M2 TAMs include PLX3397, in combination with an anti-PD-1 (pembrolizumab) and an anti-CSF-1R (cabiralizumab) antibody, in combination with the anti-PD-1 mAbs nivolumab [166]. In addition, PLX3397, in combination with other therapies, such as paclitaxel, showed significant clinical activity in advanced ovarian cancer [166]. Another clinical trial is currently underway using a CSF-1R inhibitor, LY3022855, combined with an anti-PD-L1 mAb (durvalumab) or an anti-CTL-associated protein 4 mAb, (tremelimumab) [166]. Moreover, several other small molecules, including AMG820, RG7155, BLZ945, and GW2580, are being tested in clinical trials [64, 167, 171]. Moreover, emactuzumab, as monotherapy or plus paclitaxel, as well as LY3022855, plus tremelimumab or durvalumab, are among the CSF-1R antibodies investigated in patients with advanced solid tumors, each showing moderate activity in ovarian cancer patients [167]. GW2580, a CSF-1R kinase inhibitor, can suppress macrophages’ function, lower the infiltration of M2 macrophages, and protect the vascular permeability of ECs in mouse models and advanced human patients [139, 167].

It has been found that serum level of CCL2 is increased in patients with ovarian cancer [167]. Therefore, the CCL2/CCLR axis is considered as an attractive target for therapeutic interventions. Anti-CCL2 antibodies are currently being tested in clinical trials in patients with advanced stages of the disease. However, similar to other anti-TAMs strategies, anti-CCL2 antibodies are used in combination with chemotherapy drugs such as paclitaxel, carboplatin, gemcitabine, or docetaxel [30, 169]. Combining anti − CCL2 antibody therapy with chemotherapy or immunotherapy in a mouse ovarian cancer model has improved the antitumor effects [23].

Furthermore, numerous compounds, like trabectedin and bisphosphonates, have effectively depleted macrophages by inducing their apoptosis [23]. Trabectedin, a chemotherapeutic drug approved for the treatment of ovarian cancer, has been identified to be selectively toxic to TAMs leading to reduced angiogenesis in mouse tumor models [23, 93, 165]. It is also combined with PEGylated liposomal doxorubicin for the treatment of platinum-sensitive relapsed cases [30]. In addition to targeting tumor cells, Trabectedin induces apoptosis of macrophages in the tumor by activating caspase 8 through a TNF-related ligand-dependent mechanism [30, 38]. Selective killing of macrophages with trabectedin showed clinical efficacy in a combinatorial regimen in treating recurrent ovarian cancer [151]. Trabectedin combined with pegylated liposomal doxorubicin improves OS and PFS than using pegylated liposomal doxorubicin alone in recurrent ovarian cancer patients as a second-line treatment [172, 173]. In phase II clinical trial of trabectedin, a significant decrease in blood monocytes and CCL2 levels in TAMs and ovarian tumor cells was observed. Trabectedin has been found to induce caspase 8 activation, the key effector molecule of the extrinsic apoptotic pathway, thereby causing apoptosis in monocytes. Moreover, depleting macrophage density has been a key mechanism mediating the antitumor effect in preclinical models [38]. Experimentally, bisphosphonates called liposomal clodronate are used for macrophage depletion. Clodronate, when encapsulated in liposomes, leads to preferential phagocytosis by macrophages, which ultimately results in a total macrophage depletion [167]. In vivo studies show that liposomal clodronate inhibits tumor progression and angiogenesis in mouse ovarian cancer models [166, 167]. Trabectedin and clodronate liposomes have similar functions in decreasing TAMs’ density; however, the combined use of trabectedin and clodronate does not result in any synergistic antitumor effects. On the other hand, by inhibiting CCL2 production, trabectedin resulted in a longer-lasting reduction of macrophages compared to clodronate liposomes. In addition, trabectedin kills macrophages directly and inhibits the recruitment of circulating monocytes into the tumor tissue, thereby inhibiting ovarian cancer progression [139].

Furthermore, targeting TAMs by nanoparticles, such as silica and polylactic-co-glycolic acid-based anionic nanoparticles, has shown selective accumulation in mouse models of ovarian tumors. Encapsulation of miR-125b, an mRNA within hyaluronic acid nanoparticles, demonstrated M2 repolarization to M1 macrophages in a mouse model of ovarian cancer [156, 167]. Also, intraperitoneal administration of paclitaxel with HA-PEI-miR125b nanoparticles increased the antitumor effect of paclitaxel, which was mediated by the significant reduction in ascitic fluid and peritoneal VEGF levels [156]. The infusion of IRF5 mRNA and IKKβ nanoparticles reversed the immunosuppressive and tumor-promoting phenotype of TAMs, decreased the density of M2 macrophages, and reprogrammed them to M1 macrophages [139]. It has also been shown that macrophages can also function as drug carriers to enhance antitumor effects. Engineered Adriamycin, for example, can be loaded into M1 macrophages to enable tumor drug delivery via nanotube tunneling, induction of ovarian cancer cell apoptosis, and inhibition of adriamycin-induced tumor invasion. In addition, engineered adriamycin-loaded M1 macrophages specifically penetrated the tumor lesions, decreased metastasis to almost undetectable levels, and improved OS in a mouse model of ovarian cancer [139].

Expressing PD-L1 on cell surface is a strategy by which macrophages induce immunosuppression in the TME. Blocking the PD-1/PD-L1 interaction by anti-PD-L1 antibodies has shown promising results in advanced ovarian cancer patients [169]. M2-like TAMs also regulate T cell activity by influencing PD-1 [169]. PD-L1-positive TAMs are common in primary and high-grade metastatic serous ovarian cancer and their density is associated with acute aggressiveness of this type ovarian cancer [166, 169]. Moreover, PI3K/AKT and Ras-MEK-ERK signaling pathways are the main axes inhibited via triggered PD-1 [169]. The expression of PD-L1 was significantly higher in ovarian cancer compared to other cancers and was associated with poor prognoses. A clinical trial for platinum-resistant patients is underway using the anti-PD-L1 antibody Atezoliuzub combined with Bevacizumab. Another clinical trial for recurrent and advanced ovarian tumors involves investigating a combination of MEDI4736, an anti-PD-L1 antibody, with olaparib and/or cedinarib. Therefore, novel strategies with the aim to reduce immunosuppression by decreasing monocyte recruitment, and M2/M1 ratio, as well as targeting TAMs with immune checkpoint inhibitors provide attractive targets that shift the balance of innate immunity in favor of tumor cell death [166]. AZD5153, a novel BRD4 inhibitor, showed M2 macrophage repolarization and activation of CD8 + T lymphocytes in addition to sensitizing ovarian cancer cell lines to anti-PD-L1 antibodies [167].

Furthermore, blockade of CXCL12/CXCR4 pathway prolonged the survival of tumor-bearing mice and reduced tumor burden in several preclinical studies [23]. A combination of αPD-1 and Plerixafor (AMD3100), a highly specific CXCR4 antagonist, significantly enhanced the antitumor responses compared to single αPD-1 therapy. AMD3100 has been shown to downregulate CXCL12 and CXCR4 expression and promote macrophage polarization from M2 to M1 phenotype [139].

Similar to CD47 and PD-L1, the anti-phagocytic surface protein CD24 in ovarian cancer cells acts as the primary innate immune checkpoint, binding to the inhibitory receptor siglec-10 on TAMs’ surfaces to send out a “don't eat me” signal, thereby helping cancer cells escape from the immunity. Tumors lacking CD24 expression have been found to be more sensitive to CD47 blockers, suggesting that simultaneous targeting of CD24 and CD47 may offer better clinical prospects for treating ovarian cancer [139]. Moreover, EGF secreted by TAMs, causes EGFR activation in tumor cells, which upregulated the VEGF/VEGFR signaling pathway in the surrounding, thus stimulating the proliferation and migration of cancer cells. So, blocking EGFR or intercellular adhesion molecule-1 (ICAM1) antibody neutralization in TAMs decreased cancer cell migration in mice [160].

The NF-κB signaling pathway has also been identified as a significant inducing factor for ovarian cancer invasion, progression, and chemoresistance [167, 169]. Inhibiting NF-κB in a syngeneic mouse model led to increased protumorigenic M2 macrophages, upregulation of protumorigenic soluble factors, such as VEGF in ascites, and enhanced infiltration of M2 macrophages into the TME [166]. STAT6 is a transcription factor required for M2 polarization, and IKKβ is an upstream activator of NF-κB. M2 to M1 repolarization occurred both in vivo and in vitro when using a combined IKKβ siRNA and a STAT6 inhibitor delivered within a pH-sensitive micellar compound. Methotrexate coupled dendron nanoparticles showed TAM depletion, decreased angiogenesis, and cancer stem cells in mouse ovarian cancer models [167]. These results show that activating NF-κB in TAMs, and not in tumor cells, could be considered as a viable therapeutic strategy [166]. Some strategies using inhibition of NF-κB caused repolarization of TAMs to an antitumor phenotype, restricting tumor growth in ovarian carcinoma [174]. Inhibiting the expression of GADD45β, the NF-κB effector molecule, restored the activation of proinflammatory TAMs and promoted immune infiltration in ovarian adenocarcinoma. Therefore, GADD45β-targeted therapy can reprogram TAMs and overcome immunosuppression and T cell deprivation in the TME [139].

Moreover, the results obtained from in vitro studies on different cell lines show that TNF-α is an essential player in the regulation and activation of NF-κB in tumor cells, where TAMs are one of the sources of these cytokines in the ovarian TME. Although classical M1 macrophages generally secrete TNF-α, M2-like TAMs express the CD163 marker and produce TNF-α together with TGF-β and IL-10. TGF-β enhances the invasiveness of ovarian cancer cell lines by inducing the production and activation of various MMPs in vitro. IL-6 is another cytokine secreted by TAMs that activates the STAT3 pathway, which is required for the motility, migration, survival, and proliferation of ovarian cancer cells. In addition, using TNF-α, macrophages significantly increase the expression of the signaling proteins PI3K and Akt to promote migration and invasion abilities in ovarian cancer cell lines in vitro [169, 175]. Moreover, enhanced IL-4 signaling from mouse ovarian cancer ID8 cells increased the expression of the PI3K pathway and induced the M2 phenotype. Human epididymis protein 4 (HE4), which is upregulated in ovarian cancer cells and correlated with poor clinical prognosis, has been shown to increase M2 TAM recruitment within malignant ascites in mouse models [167].

TLR 7/8 agonists are other immunostimulatory therapeutics capable of repolarizing macrophages to an M1 state; however, TLR 7/8 agonists have poor pharmacokinetic parameters and significant off-target effects. For example, giant anionic liposomes loaded with the TLR 7/8 agonist, resiquimod, target and repolarize TAMs to an M1 phenotype when administered intraperitoneally in mouse models of ovarian cancer [167]. By using a nanobody that targets the macrophage mannose receptor, a surface marker of immunosuppressive TAMs, researchers have managed to limit off-target effects. For example, Imidazoquinoline (IMDQ), a TLR 7/8 agonist, was conjugated with a nanobody to target only the TAMs of interest instead of causing a global macrophage response. This imidazoquinolinone-nanobody conjugate successfully reprogramed M2 TAMs to an M1 state in mouse models [176]. Many studies have proved that nanoparticles can be used as an effective and suitable TAM-targeted strategy. Hyaluronic acid-coated, mannan-conjugated MnO_2_ nanoparticles (Man-HA-MnO_2_ NPs), for example, caused the repolarization of TAMs into an antitumoral M1 phenotype that significantly increased tumor oxygenation. Simultaneously, the levels of HIF-1α and VEGF were decreased, leading to further relief of tumor hypoxia, thus increased chemotherapy response. In addition, doxorubicin bound to Man-HA-MnO_2_ NPs could synergistically check the growth and proliferation of cancer cells. According to the study by Penn et al., G5-dendrimer nanoparticles loaded with methotrexate, as a ligand and toxin, selectively target the folate receptor 2, which is upregulated in ovarian TAMs, leading to TAMs depletion in cancer tissues [79].

In addition, ovarian tumor is TREM2 rich; therefore, it may benefit from anti-TREM2 mAb treatments. TREM2 protein is primarily expressed on TAMs compared to other analyzed myeloid populations, and of all the analyzed markers, ovarian cancer is most enriched in TREM2 and TAMs. Further analysis revealed worse recurrence-free survival (RFS) in those with the highest TREM2 expression. These data are consistent with TREM2 + TAMs being key mediators in developing an immunosuppressed TME as disease grade worsens. This observation that mice treated with anti-TREM2 antibody showed reduced tumor burden, suggests that individuals with highly immunosuppressive TREM2 + TAM-rich TME, like those with ovarian cancer, could benefit from administration of an antibody targeting TREM2-expressing TAMs [177].

Ovarian adenocarcinoma cell fragments produced by chemotherapy can induce the release of large amounts of proinflammatory cytokines from macrophages. They can also stimulate COX2 upregulation, which promotes tumor progression. PTUPB, a COX2 inhibitor and soluble epoxide hydrolase, can arrest the production of cytokines and lipids, normally releasing by TAMs after chemotherapy, and inhibit ovarian cancer cells’ growth induced by tumor fragments [139]. Preclinical mouse models and clinical trials have provided promising data for combining chemotherapy and TAM-blocking agents, opening the horizon for the use of integrative approaches in treating ovarian cancer. For example, administration of Docetaxel, in combination with BLZ945, a CSF-1R inhibitor, significantly prevented tumor growth, decreased TAMs’ density, increased CD8 + T cell infiltration, and inhibited lung metastasis in mouse models of EOC [156, 178].

### Skin *cancer*

TAMs are present in different kinds of skin tumors including melanoma, squamous cell carcinoma (SCC), extramammary Paget's disease (EMPD), Merkel cell carcinoma (MCC), basal cell carcinoma (BCC), mycosis fungoides (MF) and angiosarcoma [179–185]. Various strategies can be utilized as TAM-related targeted treatments for skin malignancies. Cancer-related macrophages make neovascularization by delivering angiogenetic factors like VEGF, PDGF, TGF-β), and MMPs [186].

Different studies have shown that VEGF and MMPs play a key role in skin cancers, so they can be therapeutic targets in skin cancer. In angiosarcoma, for instance, TAMs express MMP9, which may be a target of amino bisphosphonates [187]. Another report showed that inhibition of the VEGF/VEGFR pathway inhibits M2 polarization in TAMs leading to reduced vascular density and tumor growth in MCA205 mouse sarcoma. Tian et al. reported that tripartite motif protein 59 (TRIM) reduces tumor-initiating effect of TAMs in melanoma cells through inhibition of MMP9 expression [188]. They concluded that TRIM59 in TAMs could be a potential regulator of tumor metastasis, thus providing a target for immunotherapy.

A report suggests that TAMs stimulated by tumor stromal factors may play a role in skin cancer carcinogenesis. Periostin (POSTN), for example, is highly expressed in the tumor stroma of myelofibrosis and dermatofibrosarcoma protuberans (DFSP). Periostin increases the production of MMP1 and MMP12 by immature M2 macrophages [189]. In another report, it was shown that POSTN and IL-4-producing MF stromal cells can stimulate TAMs to produce chemokines associated with MF tumorigenesis [188] and thus, TAMs can be modified by chemokines inhibited by immunomodulatory agents such as IFN-α and IFN-γ leading to their therapeutic effects [190].

POSTN is expressed in metastatic melanoma (14). In addition, TAMs are abundant in the tumor stroma in melanoma [181, 189, 191]. POSTN causes the stimulation of CD163 + macrophages to produce several cytokines including Treg-associated chemokines [chemokine ligand 17 (CCL17), CCL22] [180] which in turn induce the recruitment of Tregs to the tumor site in melanoma [181, 192, 193]. Thus, the repolarization of TAMs by IFN-β and imiquimod is useful for suppressing tumor growth in melanoma. Also, in B16F10 melanoma mice that were treated with cytotoxic antimelanoma drugs such as dacarbazine, nimustine hydrochloride, and vincristine, the production of CCL22 was reduced [181, 192]. Therefore, POSTIN may be a target for molecular targeted therapy in the future. Receptor activator of NF-κB (RANK) ligand (RANKL) is expressed in skin cancers of apocrine origin such as EMPD and apocrine carcinoma [180, 187] and is released in its soluble form. Monocyte-derived M2 macrophages produce MMP1 and MMP25 by stimulating RANKL.

In EMPD, soluble RANKL released by Paget cells increases the expression of CCL5, CCL17, and CXCL10 from polarized RANK + M2 TAMs, suggesting that Paget cells can modulate the immune microenvironment by stimulating TAMs. The results of this study led to the hypothesis that denosumab, a fully human mAb against RANKL, has therapeutic effects in aggressive EMPD [182, 194].

TAMs also increase the levels of cytokines by stimulating other stromal cells such as fibroblasts [195, 196]. A study by Yang et al. reported that IL-1β produced by TAMs stimulates fibroblasts to produce CXCR2 ligand, which plays an important role in the recruitment of granulocytic MDSCs to tumor sites [196, 197]. The authors concluded that CXCR2 agonists in combination with anti-CD115 antibodies could suppress B16F10 melanoma in vivo by inhibiting the recruitment of granulocytic MDSCs and reducing immature TAMs. Interestingly, emactuzumab, an anti-human CD115 mAb, reduced the number of CD163 + CD206 + M2 macrophages in melanoma patients by depleting immature TAMs before the IL-4-stimulated phase [198]. These reports imply that anti-CXCR2 agonists in combination with emactuzumab may induce an antimelanoma immune response by decreasing the number of M2 polarized TAMs.

Repolarization from M1 TAMs toward an inflammatory M2 TAMs is an effective way for the treatment of melanoma, which exhibit high glycolytic activity, leading to increased acidosis in the TME. It has been shown that inhibition of tumor acidosis by adenylyl cyclase inhibitor MDL-12 induces NOS, CXCL9, CXCL11, and TNF-α-expressing proinflammatory TAMs that are phenotypically similar to classical M1 macrophages, leading to suppression of tumor growth in B16 mouse melanoma [199]. As TAMs constitute the immunosuppressive microenvironment at the tumor site, they may be great therapeutic targets in cancer. Fujimura et al. showed the immunomodulatory effects of antimelanoma drugs including dacarbazine, nimustine hydrochloride, and vincristine, on TAMs both in vitro and in vivo by inhibition of STAT3 signals [191]. Gordon et al. reported that in vivo blocking of PD-1/PD-L1 leads to increased macrophage phagocytosis, reduced tumor growth, and prolonged survival of macrophages [200].

### Colorectal *cancer*

CRC is the third most common cancer worldwide and the second cause of cancer-related mortality worldwide [120]. As one of the significant parts of TME due to their role in facilitating tumor proliferation, invasion, migration, and angiogenesis, suppression of antitumor immunity, drug, regulation of metabolism and communications with the microbiota, TAMs have an extraordinary potential for being targeted for CRC treatments. New remedial methodologies have focused on TAMs in CRC include obstructing monocyte penetration, macrophage polarization, and immunotherapy [201].

One strategy to treat primary tumors is to block the infiltration of mononuclear cells into tumor-associated inflammatory tissues. It was shown that TAMs in the hypoxic TME in colon cancer increase the expression of transcription factors HIF-1α, CXCL12 and CXCR4. Therefore, the blockade of HIF-1α/CXCR4 pathway by targeting TAMs remains a promising therapeutic strategy [202]. Studies have shown that NT157, an inhibitor of IGF1R-IRS1/2 inhibits the expression of cytokines, chemokines, and growth factors including IL-6, IL-11, IL-23, CCL2, CCL5, CXCL7, CXCL5, intercellular adhesion molecule-1 (ICAM1) and TGF-β, which inhibits TAM [203]. Mantovani et al. found that TAMs derived from monocytes can differentiate in colon cancer. Therefore, combination therapy with the ability to block differentiation is necessary to effectively target these cells. TNF-γ can induce monocyte or macrophage recruitment to the TME and inhibit their differentiation into TAM in vivo [204].

M1 macrophages act as tumor suppressors, while M2 macrophages promote CRC. In CRC, polarization of TAMs is mainly modulated by NF-κB, STAT3, WNT5A, and PI3K pathways. Furthermore, the M2 polarization of TAMs is reversible due to the characteristic plasticity of these cells. For example, Georgoudaki et al. tried to inhibit macrophage receptor expression, repolarize TAMs to M1 type, and induce antitumor activity in mouse colon cancer model MC38 [205]. Olson et al. found that tasquinimod targets myeloid cells in early stages of tumor infiltration and induces a phenotype shift from a proangiogenic and immunosuppressive phenotype (M2 type) to a proinflammatory phenotype (M1 type) that alters the TME to promote immunity, inhibit angiogenesis and metastasis [206] As tumor suppressors, T2 RNases can inhibit growth development in vivo. Furthermore, the study by Halama, N. et al. confirmed that inhibition of CCR5 can repolarize TAMs from M2 to M1 through regulation of the STAT3/SOCS3 signaling pathway in TAMs, showing the anticancer effects in patients with liver metastases from CRC [207]. Long noncoding RNAs (lncRNAs) are used as both noninvasive biomarkers and targeting molecules in CRC [208]. For example, lncRNA RPPH1 secreted by CRC cells mediates M2 polarization and induces tumor metastasis [209]. Intestinal microflora, an important regulator of the intestinal microenvironment, has been shown to modulate lncRNA gene expression in different tissues [210]. In addition, microflora can also prompt polarization of the M0 to M2 phenotype by secreting cathepsin K (CTSK), which binds to TLR4, and activates the mTOR pathway [208]. The specific CTSK inhibitor odanacatib is prescribed to inhibit tumor-associated effects and improve the prognosis of CRC patients [208]. In addition to conventional drugs recently developed, researchers are exploiting nanoparticles that release inflammatory molecule, Ru @ ICG-BLZ NP, which has high CRC specificity and low toxicity. CSF-1R kinase inhibitor BLZ945 can repolarize TAM into M1 type, thereby showing antitumor impact, which provides a new avenue for clinical translation of nanomedicine [211].

TA-targeted Immunotherapy is another strategy for cancer therapy which mainly includes checkpoint inhibitors, T cell therapy, and vaccines against tumors [200, 212, 213]. Among these approaches, immunotherapy targeting immune checkpoint inhibitors has been clinically validated against relevant targets such as CTLA4, PD-1, and PD-L1. Korehisa et al. reported that in colon cancer patients with high microsatellite instability, PD-L1 is mainly expressed on invasive frontal tumor cells and CD68/CD163-positive M2 macrophages and that PD-L1 expression is associated with poor tumor differentiation, and lymphatic invasion [213].

Programmed cell death protein 1 (PDCD1) is a receptor for immune checkpoints [214]. In CRC patients with high microsatellite instability, PDCD1 is highly expressed in M2 macrophages in the invasive zone of the CRC [213–216]. The elevated PDCD1 expression of the M1 phenotype, leading to TAM degradation and phagocytosis, is positively correlated with CRC disease status [200]. PDCD1 blockers can increase the phagocytic capacity of macrophages, thereby prolonging the patients' survival rate. This observation confirms that PDCD1 therapy can directly affect TAMs [217]. Furthermore, patients with increased infiltration of M2 macrophages in the lesion areas have the potential to achieve better efficacy. For conventional cell therapy, the antitumor efficacy of a combination of tumor-targeted antimesothelin CAR-T cells and M2 inhibitors has been confirmed and TAM-associated cell therapy based on specific markers, such as CD40, is being studied [218]. The OVA vaccine in cell models stably expressing ovalbumin peptide (OVA) can limit tumor growth by reducing the density of TAMs in CRC tissue and the use of the neutralizing antibody VEGFC / VEGFR3 can inhibit the chemotaxis of macrophages [219].

## Conclusion

This study provides an overview of our current understanding of the crosstalk between TAMs and tumor cells and comes up with new avenues regarding the potential of TAMs as novel targets for cancer therapy. Transition of TAMs from M1-type to M2-type is associated with a tumor-stimulating phenotype, which arouses tumor outgrowth and facilitates the dissemination of tumor cells. Therefore, hindering this transition, as well as depletion of M2-type TAMs in TME and reprogramming of M2-type TAMs to M1-types are some intriguing perspectives for macrophage-targeted anticancer therapy. However, the successes attained via the current therapeutic studies is far from satisfactory, because the efficacy of most of the existing strategies have proven just in vitro or in animal models and there is still a paucity for human studies. In addition, the studies have mainly focused on depletion of M2-type TAMs and hindering their stimulatory effects on tumor cells themselves. Prosperous outgrowth and metastasis of tumors is also highly influenced by other non-malignant stromal cells of the TME and the interaction of TAMs with these non-malignant cells (e.g. fibroblasts, adipocytes, pericytes, endothelial cells etc.) remains to be well elucidated.

## Data Availability

All data are published within this manuscript.
